# Polymorphic tandem repeats influence cell type-specific gene expression across the human immune landscape

**DOI:** 10.1101/2024.11.02.621562

**Published:** 2025-04-09

**Authors:** Hope A. Tanudisastro, Anna S.E. Cuomo, Ben Weisburd, Matthew Welland, Eleanor Spenceley, Michael Franklin, Angli Xue, Blake Bowen, Kristof Wing, Owen Tang, Michael Gray, Andre L.M. Reis, Jonathan Margoliash, Nehir E. Kurtas, Jeffrey M. Pullin, Arthur S. Lee, Harrison Brand, Michael Harper, Katalina Bobowik, Michael Silk, John Marshall, Vivian Bakiris, Bindu Swapna Madala, Caitlin Uren, Caitlin Bartie, Anne Senabouth, Harriet Dashnow, Liam Fearnley, Alejandro Martin-Trujillo, Egor Dolzhenko, Zhen Qiao, Stuart M. Grieve, Tung Nguyen, Eyal Ben-David, Ling Chen, Kyle Kai-How Farh, Michael Talkowski, Stephen I. Alexander, Owen M. Siggs, Leonhard Gruenschloss, Hannah R. Nicholas, Jennifer Piscionere, Cas Simons, Chris Wallace, Melissa Gymrek, Ira W. Deveson, Alex W. Hewitt, Gemma A. Figtree, Katrina M. de Lange, Joseph E. Powell, Daniel G. MacArthur

**Affiliations:** 1 Centre for Population Genomics, Garvan Institute of Medical Research, and UNSW Sydney, Sydney, NSW, Australia; 2 Centre for Population Genomics, Murdoch Children’s Research Institute, Melbourne, VIC, Australia; 3 Faculty of Medicine and Health, University of New South Wales, Sydney, NSW, Australia; 4 Faculty of Medicine and Health, University of Sydney, Sydney, NSW, Australia; 5 Garvan-Weizmann Centre for Cellular Genomics, Garvan Institute of Medical Research, Sydney, NSW, Australia; 6 UNSW Cellular Genomics Futures Institute, University of New South Wales, Sydney, NSW, Australia; 7 Program in Medical and Population Genetics, Broad Institute of MIT and Harvard, Cambridge, MA, USA; 8 Analytic and Translational Genetics Unit, Massachusetts General Hospital, Boston, MA, USA; 9 Menzies Institute for Medical Research, University of Tasmania, Hobart, TAS, Australia; 10 Department of Ophthalmology, Royal Hobart Hospital, Hobart, TAS, Australia; 11 Centre for Eye Research Australia, University of Melbourne, East Melbourne, VIC, Australia; 12 Charles Perkins Centre, The University of Sydney, Sydney, NSW, Australia; 13 Kolling Institute of Medical Research, Royal North Shore Hospital, Sydney, NSW, Australia; 14 Genomics and Inherited Disease Program, Garvan Institute of Medical Research, Sydney, NSW, Australia; 15 Department of Medicine, University of California San Diego, CA, USA; 16 Massachusetts General Hospital, Boston, MA, USA; 17 The Broad Institute of MIT and Harvard, Cambridge, MA, USA; 18 MRC Biostatistics Unit, University of Cambridge, UK; 19 Department of Biomedical Informatics, University of Colorado Anschutz Medical Campus, Aurora, CO, USA; 20 Population Health and Immunity Division, The Walter and Eliza Hall Institute of Medical Research, Parkville, VIC 3052, Australia; 21 Department of Medical Biology, University of Melbourne, Parkville, VIC, Australia; 22 Bruce Lefroy Centre, Murdoch Children’s Research Institute, Parkville, VIC, Australia; 23 Department of Genetics and Genomics Sciences and Minidich Child Health and Development Institute, Icahn School of Medicine at Mount Sinai, New York, NY, USA; 24 Pacific Biosciences of California, Menlo Park, CA, USA; 25 Imaging and Phenotyping Laboratory, Charles Perkins Centre, Faculty of Medicine and Health, The University of Sydney, Australia; 26 Lumus Imaging, St George Private Hospital, Kogarah NSW 2217; 27 Illumina Artificial Intelligence Laboratory, Illumina, San Diego, CA, USA; 28 Centre for Kidney Research, Kids Research Institute and the Department of Nephrology, The Children’s Hospital at Westmead, Westmead, NSW, Australia; 29 Cambridge Institute of Therapeutic Immunology & Infectious Disease, University of Cambridge, UK; 30 Department of Computer Science and Engineering, University of California San Diego, CA, USA

## Abstract

Tandem repeats (TRs) - highly polymorphic, repetitive sequences dispersed across the human genome - are crucial regulators of gene expression and diverse biological processes, but have remained underexplored relative to other classes of genetic variation due to historical challenges in their accurate calling and analysis. Here, we leverage whole genome and single-cell RNA sequencing from over 5.4 million blood-derived cells from 1,925 individuals to explore the impact of variation in over 1.7 million polymorphic TR loci on blood cell type-specific gene expression. We identify over 62,000 single-cell expression quantitative trait TR loci (sc-eTRs), 16.6% of which are specific to one of 28 distinct immune cell types. Further fine-mapping uncovers 4,283 sc-eTRs as candidate causal drivers of gene expression in 13.6% of genes tested genome-wide. We show through colocalization that TRs are likely mediators of genetic associations with immune-mediated and hematological traits in over 700 genes, and further identify novel TRs warranting investigation in rare disease cohorts. TRs are critical, yet long-overlooked, contributors to cell type-specific gene expression, with implications for understanding rare disease pathogenesis and the genetic architecture of complex traits.

## Introduction

Recent advances in sequencing and genotyping tools have extended our understanding of multiple classes of human genetic variation that were previously challenging to characterize. Among these, tandem repeats (TRs) - highly polymorphic repetitive sequences characterized by 1–6 bp motifs (short tandem repeats [STRs]) and >6bp motifs (variable number tandem repeats [VNTRs]) - have emerged as a key component of genetic diversity with profound biological effects^1^.

More than 2 million polymorphic TR loci comprise 3–5% of the human genome, collectively occupying more genomic real estate than protein coding genes^2,3^. Individual TRs are up to 10^4^ times more mutable than individual single nucleotide variants (SNVs)^4^, making them a rich source of genetic variation. TRs can influence gene expression by regulating transcription factor binding^2^, altering nucleosome occupancy^5^, and modulating the activity of promoters^6^ and enhancers^7^. Despite their clear functional importance, TRs have largely been understudied in population genetics, primarily due to high error rates associated with sequencing and aligning repetitive regions that have historically limited the accuracy of TR genotyping^1^. However, methodological improvements have begun to address these challenges, enabling more precise and comprehensive characterization of TRs^1^.

Rare repeat expansions have been known for several decades^8^ to cause severe Mendelian disorders, such as Huntington’s disease and Fragile X Syndrome, but emerging evidence suggests that common TR variation is an underexplored source of genetic variation that may help account for the heritability of complex traits. Recent studies have uncovered associations between common TR variation and a diverse array of phenotypes, including gene expression^9^, epigenetic modification^10^, serum biomarker levels^11^, cancer susceptibility^12,13^, and other complex disease traits^14^. However, the lack of harmonization in locus definitions^3^ across studies presents a significant challenge, making it difficult to compare results and draw conclusions about the functional impact of TR variation. Some studies^11,15^ have also relied on imputation to infer TR genotypes from SNV genotypes which, while useful, is not as reliable as direct genotyping, particularly given that highly polymorphic loci such as TRs are less likely to be in strong linkage disequilibrium with neighboring SNVs.

Expression quantitative trait loci (eQTL) studies play a crucial role in understanding how genetic variation influences biological processes by identifying genetic drivers of variation in gene expression. While studies of how TRs regulate gene expression have primarily relied on bulk tissue data^3,9^, which averages the expression of genes across heterogenous cell populations, recent advances in single-cell RNA sequencing (scRNA-seq)^16^ enable the study of cell type-specific effects of TRs at a finer resolution at population scale. Single-cell analysis provides insight into cellular heterogeneity^17^, differentiation pathways^18^, and disease progression^19^. Long-read scRNA-seq of striatal projection neurons was instrumental in uncovering the key role that somatic TR expansions play in the progression of Huntington’s disease^20^, subsequently opening new avenues for gene therapy. Integrating TRs in single-cell analyses promises deeper insights into the mechanisms of known TR-associated phenotypes, and may reveal novel functions of these repetitive sequences.

Here we present a catalog of single-cell expression quantitative trait TR loci (sc-eTRs) derived from peripheral blood samples in Phase 1 of TenK10K, the largest paired human whole genome and single-cell RNA sequencing dataset to date^21^. We identify TRs robustly associated with variation in gene expression in at least one cell type, and characterize their cell type specificity, evidence for causality, and potential colocalization with signals previously shown to be associated with a variety of immune and hematological traits.

## Results

### Genome-wide polymorphic TR discovery

**Figure 1:**
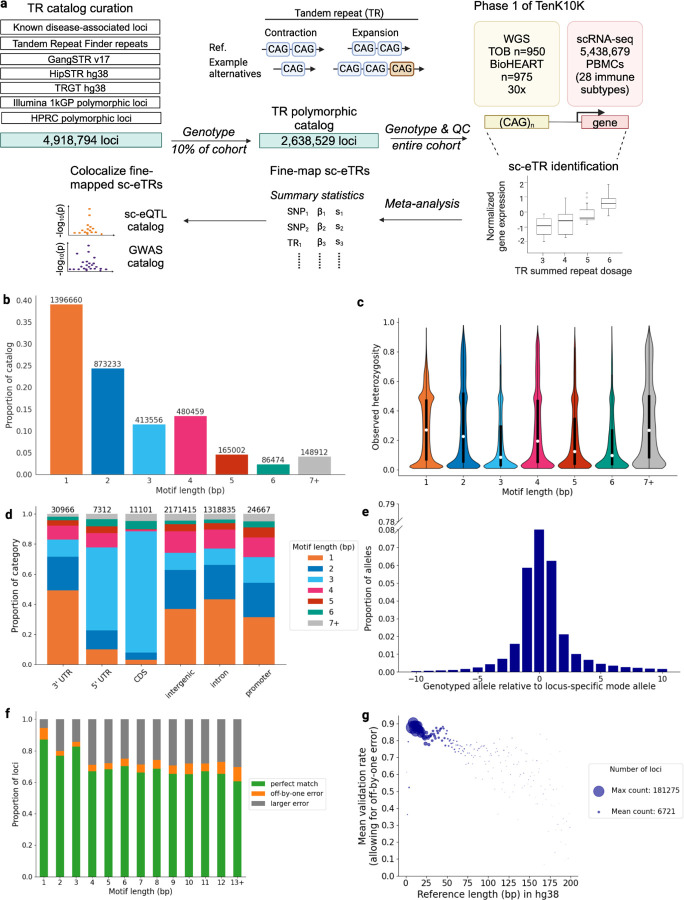
Genome-wide polymorphic TR curation and validation. **a,** Schematic of study design involving TR catalog curation, sc-eTR identification, fine-mapping, and colocalization analysis. Tasmanian Ophthalmic Biobank cohort is abbreviated as TOB and peripheral blood mononuclear cells as PBMCs. **b**, Motif length distribution of TR loci in the polymorphic catalog. **c**, Observed heterozygosity of TR loci in the polymorphic catalog, by motif length. Violin plots summarize the distribution of locus-specific observed heterozygosity rates, showing median values (white circle) and boxes spanning from the 25th (Q1) to the 75th percentile (Q3). Whiskers extend to Q1 – 1.5 x IQR and Q3 + 1.5 x IQR, where IQR is the interquartile range. **d**, Gene annotation (MANE) of TR loci in the polymorphic catalog, by motif length. **e**, Distribution of genotyped alleles relative to the locus-specific modal allele, restricted to genotyped alleles within 10 repeats of the mode allele. **f**, Validation of ExpansionHunter genotypes using TRGT genotypes derived from PacBio HiFi long read samples (n=25) across 1,745,049 TR loci used in downstream sc-eTR discovery, by motif length. Perfect match calls were those where the ExpansionHunter summed repeat dosage exactly matched that of TRGT, off-by-one error were those where the ExpansionHunter summed repeat dosage was +/− 1 repeat from that of TRGT, with the remaining discordant calls labeled as ‘larger error’. **g**, Mean validation rate (allowing for off-by-one error) of ExpansionHunter genotypes using TRGT genotypes (n=25 samples), by reference length (bp) of the TR locus. Horizontal axis is truncated to 200 bp, capturing more than 99.9% of the loci.

To investigate the relationship between TR length variation and gene expression across 28 immune cell types, we first identified polymorphic TR loci in 1,925 individuals from Phase 1 of the TenK10K initiative for whom both high-coverage whole genome sequencing (WGS) and single-cell RNA sequencing (scRNA-seq) data from over 5.4 million peripheral blood mononuclear cells (PBMCs) were available (Fig. 1a). The 1,925 individuals analyzed in this study consists of individuals from the Tasmanian Ophthalmic Biobank cohort (950 European ancestry individuals with no signs of ocular disease) and the BioHEART cohort (975 European ancestry individuals with diagnosed or suspected cardiac disorders) (Methods).

Most TR genotyping tools require target loci to be specified *a priori*, meaning that catalog curation is crucial for sensitivity. To generate a genome-wide catalog of candidate TRs, we merged several reference-based (hg38)^22–25^ and cohort-driven polymorphic TR catalogs, using samples from the 1000 Genomes Project^26^ and the Human Pangenome Reference Consortium^27^, generating a final catalog of 4,918,794 loci^28^ (Methods).

To curate a set of loci likely to be sufficiently polymorphic to drive an association signal, we then used ExpansionHunter to genotype these loci in approximately 10% of the cohort, identifying 2,638,529 polymorphic loci for downstream analysis. Homopolymer and dinucleotide motifs comprised the majority of the polymorphic loci, consistent with other polymorphic catalogs^27,29^ (Fig. 1b). Polymorphism, measured as per-locus observed heterozygosity, was lowest in tri- and hexanucleotide motifs, consistent with these motifs having lower reported mutation rates^30^ (Fig. 1c). Based on Matched Annotation from NCBI and EBI (MANE)^31^, most polymorphic loci were located in intergenic (60.9%) and intronic (37.0%) regions, with others found in promoter (0.69%), coding sequence [CDS] (0.31%) 3’ untranslated region [UTR] (0.87%), and 5’ UTR (0.21%) regions (Fig. 1d).

Because TR genotyping is prone to stutter and remapping errors, we benchmarked the accuracy of three short-read TR genotyping tools - ExpansionHunter^32,33^, HipSTR^24^, and GangSTR^23^ - by comparing their WGS-derived genotypes with gold standard capillary electrophoresis-derived genotypes of 388 STR loci in 120 samples from the Simons Genome Diversity Project^34^ (Fig. S1, Methods). ExpansionHunter had the best performance with a validation rate of 95.0%, followed by HipSTR and GangSTR (94.9%, 87.4% respectively). The performance of ExpansionHunter and HipSTR are comparable to prior validation studies^24,33^.

We used ExpansionHunter to genotype TRs in the rest of the cohort, and found that 97% of raw genotyped alleles were within 10 repeats of the modal allele of each locus, consistent with a stepwise mutation model^35^ (Fig. 1e).

We subsequently filtered for high quality calls and loci for downstream analysis (Methods). Using approximately 1.7 million filtered loci, we further validated the accuracy of ExpansionHunter with PacBio HiFi long-read TR genotypes (n=25 individuals), reporting a strict genotype concordance rate of 80.9%, which increased to 86.5% when allowing for +/− 1 repeat unit difference (Fig. 1f, Methods). As expected, validation rate tended to decrease as TR reference length increased (Fig. 1g). Notably, this PacBio genome-wide approach included TRs in low complexity regions, in contrast to the capillary electrophoresis validation dataset described above where TR loci were flanked by non-repetitive sequences and thus had improved read mappability (Note S1, Fig. S2), thus providing a more unbiased genome-wide estimate of error rates. While these error rates are (as expected) substantially higher than those seen for more simple variant classes such as SNVs, such errors are expected to reduce our sensitivity in the associations reported below rather than to create false positive associations.

### Characterization of single-cell expression TRs (sc-eTRs) across 28 immune cell types

We identified *cis* sc-eTRs (*i.e.* eQTL associations with TR variants close to the gene of interest) by testing associations between 1,745,049 common autosomal TRs (non-major allele frequency ≥ 0.5%) and the expression of 22,213 genes across 28 immune cell types. We defined an sc-eTR as an eTR/eGene pair association detected in a given cell type. Using associaTR, we performed pseudobulk eQTL mapping within 100 kb of each gene’s transcription start and end sites, controlling for technical and biological covariates (Fig. 1a, Methods). We conducted parallel analyses for common single nucleotide variants (SNVs) (minor allele frequency ≥ 1%) and employed a two-batch meta-analysis approach to mitigate cohort-specific sequencing effects while accounting for effect size heterogeneity (Fig. S3, Methods).

**Figure 2:**
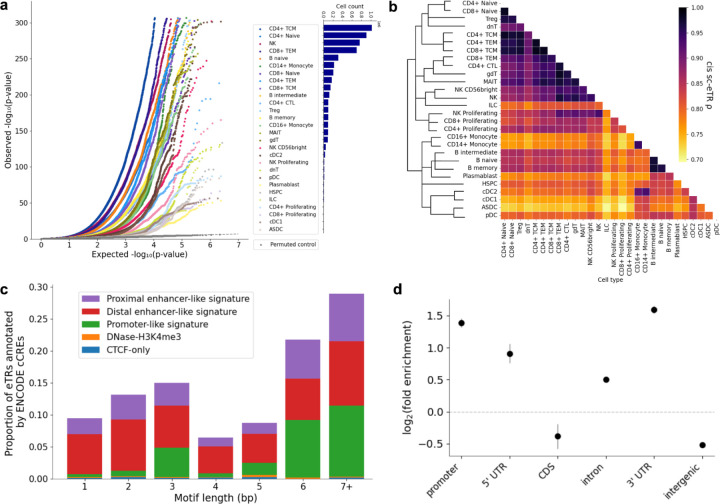

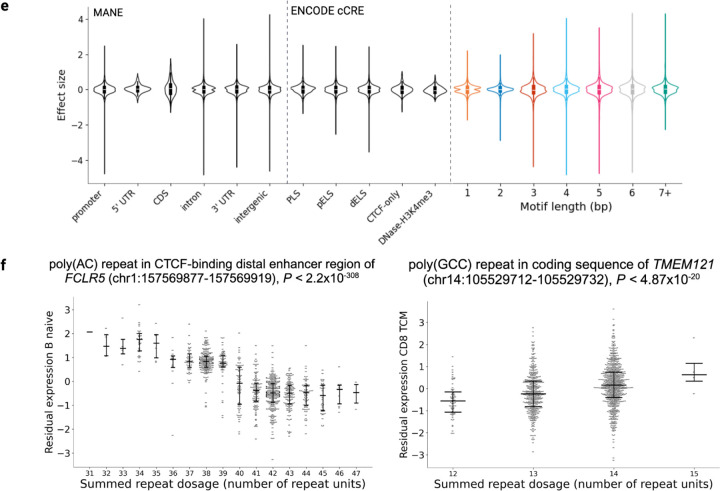
single-cell eTR discovery. **a,** Quantile-quantile (QQ) plot comparing observed *P* values for each TR x gene test (colored by cell type) against the expected uniform distribution for each cell type (dashed gray line). Permuted control is highlighted in dark gray (n=1,925). **b**, Heatmap of pairwise correlations of *cis* sc-eTR posterior effect sizes across cell types, adjusted for global effect size sharing and power differences with multivariate adaptive shrinkage^36^. Order of rows and columns reflect scRNA-seq cell type classification by scPred^38^ (Methods). **c**, ENCODE candidate *cis* regulatory element (cCRE) annotation of sc-eTRs, by motif length. **d**, Log_2_(fold enrichment) of sc-eTRs relative to all TRs genotyped, by MANE gene annotation. Error bars correspond to 95% confidence intervals. **e**, Distributions of effect sizes, by gene annotation (MANE), ENCODE cCRE, and motif length. Violin plots summarize the distribution of effect sizes with horizontal lines showing median values and boxes spanning from the 25th (Q1) to the 75th percentile (Q3). Whiskers extend to Q1 – 1.5 x IQR and Q3 + 1.5 x IQR, where IQR is the interquartile range. **f**, Examples of single-cell eTRs validated using GTEx whole blood bulk RNA-seq data^9^: a naive B cell-specific poly(AC) repeat associating with expression of *FCRL5*, which encodes an immunoglobulin receptor promoting B cell proliferation^39^; a CD8_TCM_-specific poly(GCC) coding repeat associating with expression of *TMEM121.*

We identified 62,345 unique sc-eTRs associating with 14,732 eGenes at a gene-level false discovery rate (FDR) of 5% (Fig. 2a, Fig. S4, Table S1). Type 1 error (false positive rate) was controlled, assessed by permutation (Fig. 2a, Methods). The number of sc-eTRs detected in each cell type correlated with the number of cells sequenced in each cell type (Pearson *r* = 0.921, *P* = 4.028×10^−12^), but we detected sc-eTRs in every cell type (Fig. 2a). Meta-analysis identified a greater number of sc-eTRs compared to analyses of individual cohorts, with the number of sc-eTRs discovered correlating with sample size (Fig. S5). Notably, meta-analysis identified sc-eTRs (FDR < 5%) in 200 eGenes that failed to reach significance in either cohort.

After accounting for global effect size sharing and power differences between cell types using multivariate adaptive shrinkage^36^, pairwise similarity of cell types was quantified by the correlation of the posterior effect sizes of sc-eTRs, showing patterns of cell type similarity consistent with hierarchical classification, albeit weaker for rarer cell types (Fig. 2b). More closely related cell types, such as subsets of B cells, exhibited high pairwise correlation of their effect sizes, while lymphoid effects were less concordant with those observed in myeloid cell types. The effect sizes of type-2 conventional dendritic cells (cDC2) and monocytes showed strong concordance, consistent with cDC2 sharing the most markers with monocytes amongst all dendritic cell (DC) subtypes^37^. The pairwise correlation of posterior effect sizes of sc-eTRs was comparable to that observed for single-cell expression quantitative trait single nucleotide variants (sc-eSNVs) (Fig. S6).

sc-eTRs were enriched in ENCODE candidate *cis* regulatory elements (cCREs) (odds ratio [OR] = 1.84, Fisher’s exact test, *P* < 2.2×10^−16^). Among cCRE-overlapping sc-eTRs, the majority were found in distal enhancer-like signatures (Fig. 2c). Considering genomic context, sc-eTRs were enriched in proximal regulatory regions and depleted in coding and intergenic regions (Fig. 2d). Across all sc-eTRs, increasing repeat length generally corresponded to higher gene expression (median effect size =0.016, Wilcoxon signed-rank test, *P* = 1.671×10^−3^). This positive association was most pronounced in sc-eTRs overlapping introns, 5’ UTRs, and 3’ UTRs (*P* = 1.276×10^−4^, 7.687×10^−4^ and 2.688×10^−3^ respectively, Fig. 2e). Broader effect size distributions were observed in intergenic sc-eTRs compared to those in proximal regulatory elements (Levene’s test, *P* = 1.231×10^−3^, Fig. 2e).

To determine cell type specificity of sc-eTRs, we performed pairwise meta-analyses comparing the effect sizes of sc-eTRs in each cell type against all other cell types where the respective gene was expressed in at least 1% of cells. sc-eTRs were considered shared if the meta-analysis p-value was lower than the initial p-value. Conversely, sc-eTRs were considered cell type-specific if the meta-analysis p-value was consistently higher than the initial p-value across all pairwise comparisons (Methods).

Of the 62,345 unique sc-eTRs identified, 16.6% were specific to one cell type (*i.e.* cell type-specific). Across all cell types, the median pairwise sharing of top sc-eTRs was 12.8% (minimum = 5.7%, maximum = 54.4%), suggesting the presence of cell type-specific regulatory mechanisms. Monocytes had the highest level of intra-subset sharing (median = 41.4%) while sharing within the CD8+ (median = 36.1%), NK (median = 34.4%), CD4+ (median = 27.3%), and B (median = 25.0%) cell subsets were lower. We tested for enrichment of eGenes associated with cell type-specific sc-eTRs among Gene Ontology (GO)^40–42^ terms and recapitulated known functional associations between cell types and their eGenes (Note S2).

Cell type-specific sc-eTRs had greater absolute effect sizes (Mann-Whitney U test, *P* = 3.406×10^−248^) and were, on average, located further away from the transcription start site (*P* = 8.239×10^−46^) than shared sc-eTRs, consistent with previous observations in cell type-specific eSNVs^43^. Compared to shared sc-eTRs, cell type-specific sc-eTRs were also enriched in distal enhancer-like signatures and depleted in promoter-like signatures, aligning with earlier findings in cell type-specific eSNVs^44^ (Fig. S7).

We identified 310 sc-eTRs with opposite signed effect sizes in at least two cell types, highlighting the importance of cell type context in gene expression. Increasing repeat length of poly(TAT) intronic repeat overlapping an enhancer of *PREX1* was associated with contrasting effects on *PREX1* expression (Fig. S8), markedly increasing expression in naive CD4+ cells (effect size = 0.896, *P* = 1.547×10^−81^) while reducing it in CD16+ monocytes cells (effect size = −0.396, *P* = 1.268×10^−13^). Notably, upregulation of PREX1 promotes proliferation of naive CD4+ T cells^45^, whereas its deficiency reduces macrophage chemotaxis^46^. Thus, repeat length variation in this locus may fine-tune immune responses through cell type-dependent *PREX1* expression.

We further considered eTRs previously identified in whole blood bulk RNA-seq GTEx data^9,47^ (n = 652 individuals), and found that 91.1% of them overlapped with nominally significant sc-eTRs (*P* < 0.05) in at least one cell type in our dataset (Fig. 2f, Fig. S4, Table S2, Methods). Signals unique to sc-eTRs were enriched for cell type specificity compared to those overlapping bulk eTRs identified in GTEx (OR = 1.35, Fisher’s exact test, *P* = 0.001).

### Functional enrichment and LD profiles of candidate causal sc-eTRs

**Figure 3:**
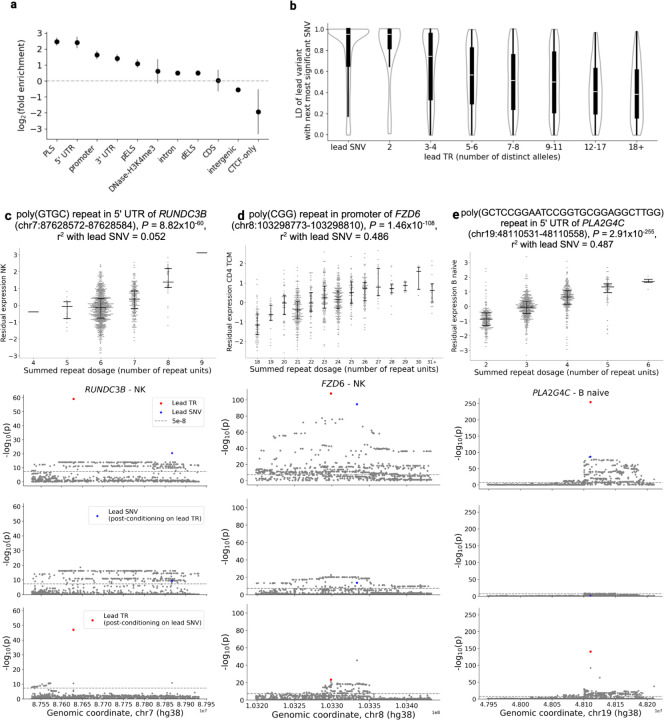
Characterization of candidate causal sc-eTRs. **a**, Functional enrichment of candidate causal sc-eTRs relative to all TRs genotyped. The y axis denotes log_2_fold enrichment. Error bars correspond to 95% confidence intervals. ENCODE candidate *cis* regulatory element (cCRE) annotations are abbreviated as follows: promoter-like signature [PLS], proximal enhancer-like signature [pELS], distal enhancer-like signature [dELS], CTCF binding site failing to intersect another cCRE annotation [CTCF-only]. **b**, Linkage disequilibrium (LD) for lead SNVs and TRs and their respective next most significant neighboring SNV (within +/− 100kb from gene body). Lead TRs are divided into equally sized bins reflecting the number of distinct alleles genotyped in the cohort. **c-e**, Case examples of candidate causal sc-eTRs. For each locus, the upper plot shows the association between repeat length and residual gene expression (with covariates regressed out), while the remaining plots show the results of the association analysis, conditional analysis on the genotype of the candidate causal sc-eTR, and conditional analysis on the genotype of the lead SNV. In detail, the second plot shows the original association signals for the candidate causal sc-eTR (red circle), the lead eSNV (blue star), and other SNVs and TRs within +/− 100kb of the gene body (gray points). The third plot shows the association signals for the same set of variants, conditioning each on the summed genotype of the candidate causal sc-eTR. The fourth plot shows the association signals for the same set of variants, conditioning each on the genotype of the lead eSNV.

Despite TRs exhibiting on average higher genotyping uncertainty than SNVs, we identified 10,172 genes where sc-eTRs were the lead variant (*i.e.* exhibited the strongest association signal within their respective *cis* windows compared to the signals of neighboring sc-eTRs and sc-eSNVs). As expected given the higher mutation rate at TR loci, lead sc-eTRs exhibited, on average, weaker LD with the next most significant neighboring SNV (proxy SNV) compared to lead SNVs in the best-powered cell type (Fig. S9). The strength of LD was inversely associated with the number of distinct alleles genotyped at the TR locus (Fig. 3b). Additionally, lead sc-eTRs tended to exhibit a larger difference in strength of association, as measured by p-value ratio, relative to their proxy SNVs than those observed for lead SNVs and their proxies (Fig. S10).

The presence of these lead sc-eTRs suggests either large effect sizes that overcome genotyping uncertainty, or a subset of TR loci with lower genotyping uncertainty. In either case, these lead sc-eTRs are strong candidates for causal drivers of gene expression variation.

From an initial set of 62,345 unique sc-eTRs, we identified 4,283 as candidate causal drivers of gene expression variation (Table S3). In each cell type, candidate causal sc-eTRs were conservatively defined as lead sc-eTRs fine-mapped with a posterior inclusion probability (PIP) ≥ 0.7. To fine-map lead sc-eTRs, we initially attempted SuSiE with 10 maximum credible sets, but identified instability in the results related to meta-analysis and imperfect representation of LD between multiallelic TRs and biallelic SNVs (Note S3, Fig. S23–24). Consequently, we performed fine-mapping with SuSiE by conservatively assuming that each locus has at most one causal variant. Candidate causal sc-eTRs were identified across 3,016 eGenes, suggesting that sc-eTRs were the candidate causal signal in 13.6% of genes tested in at least one cell type.

Candidate causal sc-eTRs were most enriched in proximal regulatory elements including cCREs with promoter-like signatures, 5’ UTRs, and promoter regions (OR = 5.664, 5.366, 3.133 respectively, Fisher’s exact test, *P* < 2.2×10^−16^ for all comparisons, Fig. 3a). These enrichments were stronger in candidate causal sc-eTRs compared to all sc-eTRs (Fig. S11). Conversely, candidate causal sc-eTRs were negatively enriched in more distal regulatory elements found in intergenic regions (OR = 0.458, Fisher’s exact test, *P* < 2.2×10^−16^, Fig. 3a). We also observed that GC-rich TRs, defined as loci where guanine and cytosine nucleotides constitute at least 80% of the motif, were enriched in candidate causal sc-eTRs compared to all genotyped TRs (OR = 1.349, Fisher’s exact test, *P* = 0.00054).

The number of eGenes associated with candidate causal sc-eTRs exhibited an inverse relationship with protein-coding constraint, quantified using the loss-of-function observed/expected upper bound fraction (LOEUF) score (Spearman’s ρ = 0.964, *P* = 7.321×10^−6^, Fig. S12); genes under strong evolutionary constraint (low LOEUF deciles) harbored significantly fewer eGenes compared to less constrained genes. This finding aligns with the expectation that dosage-sensitive genes - typically characterized by highly constrained coding sequences - face stronger selective pressure against expression-modifying variants, resulting in depletion of regulatory variation^48^.

We then considered enrichment of candidate causal sc-eTRs in non-coding regulatory constraint elements across species. By leveraging both Zoonomia^49^ and primate-specific^50^ annotations, we found that these sc-eTRs exhibited preferential enrichment for transcription factor binding sites (TFBS) within constrained primate-specific non-coding elements while showing attenuated enrichment in more deeply conserved mammalian and Zoonomia elements, aligning with previous observations of fine-mapped eSNVs^50^ (Fig. S13). Similarly, candidate causal sc-eTRs were enriched in Zoonomia Group 2 and 3 (actively evolving and primate-specific respectively) cCREs (Fig. S13).

The association between regulatory potential and primate-specific constraint showed marked GC-content dependence, consistent with TFBS and open chromatin regions being on average GC-rich^51,52^. Notably, candidate causal sc-eTRs with 61–80% GC content demonstrated the strongest enrichment for TFBS in constrained primate-specific elements (OR = 10.870, Fisher’s exact test, *P* = 0.00059). Higher GC content (81–100%) sc-eTRs exhibited substantial enrichment in DNase hypersensitivity summit sites within constrained primate elements (OR =3.027, Fisher’s exact test, *P* = 6.157×10^−7^).

TR variation has previously been reported to intersect epigenetic markers, influencing methylation patterns at nearby CpG sites^10^. We identified 189 distinct candidate causal sc-eTRs significantly associated with methylation of nearby CpG sites (within 5 kb) using methylation signatures derived from PacBio HiFi sequencing of a subset of our cohort (n=25, Bonferroni-corrected *P* < 0.05, Table S4). On average, each candidate causal sc-eTR associated with approximately 1.8 nearby CpG sites (standard error = 0.105). Compared to all TRs genotyped, candidate causal sc-eTRs associating with methylation were significantly enriched in 3’ UTR and intronic regions (OR = 2.370, 1.916, Fisher’s exact test, *P* =0.032, 1.844×10^−9^ respectively), and depleted in intergenic regions (OR = 0.508, Fisher’s exact test, *P* = 3.344×10^−10^). Repeat length was not associated with a consistent direction of methylation change (median effect size = 0.133, Wilcoxon signed-rank test, P = 0.076).

To further contextualize these findings, we colocalized eGenes associated with candidate causal sc-eTRs with methylation eQTL signals from a published dataset of SNVs and TRs^10^ (Methods). We identified 485 eGenes where methylation and single-cell gene expression signals significantly colocalized (PP H4 ≥ 0.8) (Table S5).

Among these, 36 eGenes (7%) showed direct evidence of candidate causal sc-eTRs associating with methylation in our PacBio dataset, yielding a subset of candidate causal sc-eTRs that are likely regulating gene expression through epigenetic mechanisms (Table S6). sc-eTRs associated with these 36 eGenes demonstrated marked cell type specificity, with 81.1% of them shared among at most four cell types (Fig. S14, Table S6). CD4+ naive T cells were most enriched in this set of methylated sc-eTRs (OR = 4.42, Fisher’s exact test, *P* = 0.009).

Direct genotyping of TR loci revealed several novel fine-mapped associations, with candidate causal sc-eTRs exhibiting moderate linkage disequilibrium with the lead SNV of the respective *cis* window (median r^2^ = 0.448, interquartile range [IQR] = 0.438) (Fig. S15). Approximately 45.8% of candidate causal sc-eTRs remained significant (*P* < 5×10^−8^ ) after conditioning on the genotype of the lead SNV, suggesting that a subset of these sc-eTRs may explain additional variation in gene expression beyond what can be explained by SNVs alone (Table S7).

For instance, we found a CTCF-binding poly(GTGC) 5’ UTR repeat to be candidate causal for *RUNDC3B* expression in 9 lymphoid cell types (minimum *P =* 8.816×10^−60^, Fig. 3c). Previous work has shown that *RUNDC3B* promoter methylation is specific to lymphoid cell lines, and absent in myeloid lineages^53^. As another example, we identified a poly(CCG) repeat in the promoter of *FZD6* as candidate causal for its expression in CD4_TCM_ cells (P = 1.464×10^−108^, Fig. 3d). FZD6 is involved in the Wnt signalling axis which promotes T cell development^54^. One of the strongest signals we observed was a poly(GCTCCGGAATCCGGTGCGGAGGCTTGG) repeat in the 5’ UTR of *PLA2G4C*, specific to B-cells (minimum *P* =2.908×10^−255^, Fig. 3e). PLA2G4C hydrolyses glycerophospholipids, releasing free fatty acids and other lipids to be used as precursors for signaling molecules^55^.

While rare repeat expansions in over 60 TR loci have been identified as causal for severe monogenic disorders^56^, the impact of common variation at these loci is less understood^57^. Surprisingly, given most disease-associated TRs act through neuronal pathways^8^ and are not known to have biological impact in blood cells, common TR variation was found to nominally associate with gene expression across 51 disease-associated loci (nominal *P* < 0.05). In 3 of these loci - *GLS*, *PPP2R2B*, and *FRA10AC1*- common TR variation was identified as candidate causal for gene expression (Table S8, Note S4, Fig. S25–26).

We considered how candidate causal sc-eTRs could annotate disease-associated genes lacking known TR-mediated mechanisms. We hypothesized that TR length variation could contribute to variable penetrance of pathogenic variants in *cis*, mask causative dominant variants in autosomal dominant haploinsufficient genes in *trans*, or act as a ‘second hit’ in a recessive gene with one candidate pathogenic variant. We found that 23.4% of eGenes associated with candidate causal sc-eTRs were represented in PanelApp^58^, a public database of gene panels for human disorders (Table S9), highlighting a subset of loci that warrant further exploration in severe genetic disease cohorts.

### Candidate causal sc-eTRs likely drive complex traits

**Figure 4:**
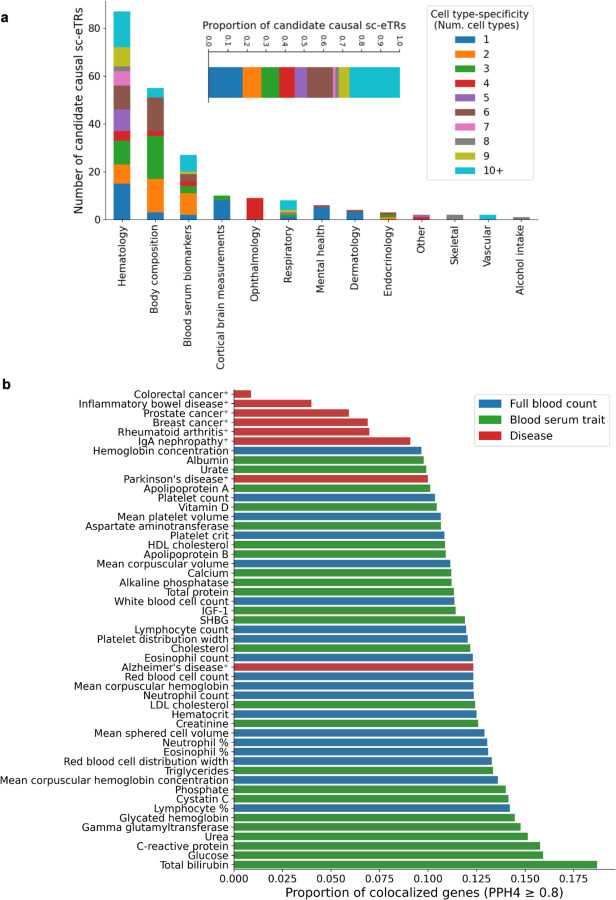
Candidate causal sc-eTRs associate with complex trait variation. **a**, Barplot of the number of candidate causal sc-eTRs intersecting TR UKBB PheWAS hits, colored by cell type specificity. **b,** Barplot of the proportion of colocalized genes (PPH4 ≥0.8) associated with a candidate causal sc-eTR relative to all genes tested for colocalization, per GWAS catalog. Phenotypes marked with ✝ were colocalized using only SNVs while unmarked phenotypes were colocalized using SNVs and imputed TRs.

We subsequently investigated whether candidate causal sc-eTRs could help explain variation in complex traits. We first intersected candidate causal sc-eTRs with significant TR PheWAS hits from UK Biobank (UKBB)^14^ (Fig. 4a, Table S10, Methods). Candidate causal sc-eTRs associating with hematological traits were enriched in 5’ UTR and promoter regions, compared to all candidate causal sc-eTRs (OR = 8.923, 3.168, Fisher’s exact test, *P* = 3.582×10^−5^ and = 0.027 respectively, Fig. S16).

We then performed colocalization analysis to compare our sc-eQTL (sc-eSNV+sc-eTR) data with existing GWAS catalogs. The goal of colocalization is to determine whether a common genetic variant is likely to influence both gene expression and a complex trait, thereby providing insights into the biological mechanisms underlying associations observed in GWAS. We performed colocalization with two types of catalogs: (1) SNV-only GWAS catalogs for autoimmune conditions (inflammatory bowel disease [IBD]^59^, rheumatoid arthritis^60^, systemic lupus erythematosus^61^, and IgA nephropathy^62^), cancer (breast^63^, colorectal^64^, lung^65^, lymphoma^66^, prostate^67^, Non-Hodgkin’s lymphoma^68^, and lymphocytic leukemia^68^), COVID-19^69^, and neuroinflammatory conditions (Alzheimer’s^70^ and Parkinson’s disease^71^); and (2) joint SNV and imputed STR GWAS catalogs for 44 UKBB blood serum and full blood count traits^11^ (Fig. 4b).

We hypothesized that candidate causal sc-eTRs could drive GWAS signals if the following conditions were met: (1) strong colocalization indicating that the trait and gene expression are driven by the same variant (PP H4 ≥ 0.8), (2) evidence that the sc-eTR is the lead variant driving changes in gene expression and is confidently fine-mapped (PIP ≥ 0.7 using a single causal variant model), and (3) evidence that the sc-eTR is in at least moderate linkage disequilibrium (LD, r^2^ ≥ 0.5) with a variant cataloged in the GWAS window.

For each trait, we restricted colocalization to eGenes that contained at least one candidate causal sc-eTR (*P* < 5×10^−8^) within 100 kb of the gene body, and that had at least one genome-wide significant (*P* < 5×10^−8^) signal within the same window in the GWAS catalog. We used coloc^72^ to compute the probability that the trait and expression of the gene were driven by the same variant (Methods).

We identified 788 distinct genes (2,183 gene x trait combinations) where the candidate causal sc-eTR was in at least moderate LD (r^2^ ≥ 0.5) with a variant in the GWAS catalog, and there was strong evidence that the trait and expression of the gene were driven by the same variant (PP H4 ≥ 0.8) (Table S11). Generally, the greatest number of colocalizations was observed for full blood count (FBC) traits, followed by blood serum traits, and finally disease phenotypes, likely reflecting both the relative order of impact of gene expression and extent of polygenic contribution to these traits (Fig. S17). Controlling for polygenic contribution, the proportion of genes tested with strong colocalization evidence (PP H4 ≥ 0.8) varied by trait, with 14.2% of loci colocalizing for lymphocyte percentage and 12.3% for Alzheimer’s disease (Fig. 4b).

As an example, we found a poly(T) candidate causal sc-eTR in the promoter of *KIF16B* strongly colocalizing with mean platelet volume in cytotoxic immune cells (CD4_CTL_, NK subtypes, CD8_TEM_, and CD8+ naive) (maximum PP H4 = 1; minimum *P <* 2.2×10^−308^) (Fig. S18). *KIF16B* encodes a kinesin motor protein, which transports intracellular signaling molecules to the cell membrane^73^. Kinesin proteins have been found to regulate pro-inflammatory α-granule and dense granule secretion in platelets^74^ and have also been found to influence lytic granule secretion in T^75^ and NK cells^76^. Our results suggest that *KIF16B* may be involved in the immunological synapse between cytotoxic cells and platelets during pro-inflammatory states^77^.

The cell type specificity of our dataset also allows for further contextualization of sc-eTRs in relation to disease. We found that a poly(CGCA) sc-eTR in the intron of *GRB10*, which overlaps with an enhancer with an activity by contact (ABC) score ≥ 0.015^78^, is significant in CD4+ naive T cells, and strongly colocalizes with prostate cancer (PP H4 = 0.962, *P* = 8.144×10^−126^). GRB10 has been established to act as an effector of PI3K and sustains andron receptor signalling in prostate cancer cells, promoting growth and proliferation^79,80^. GRB10 expression has also been found to be proportional to the population of CD4+ naive T cells^81^. The identification of this CD4+ naive T cell-specific eTR may provide mechanistic insights into GRB10 modulation in the context of prostate cancer, warranting further functional validation to assess its potential as a cell type-specific therapeutic target.

We also found a CD8+ naive-specific poly(GGA) sc-eTR in the coding sequence of *ALMS1* colocalizing with hematocrit and serum IGF-1 (PP H4 = 0.977, 0.965 respectively; P = 6.018×10^−37^) (Fig. S19). *ALMS1* codes for a centrosomal protein critical for microtubule organization^83^. Pathogenic variants in *ALMS1* cause Alström syndrome, a condition characterized by systemic features, including impaired growth hormone signaling and IGF-1 deficiency^84^.

In addition, we observed a naive B cell type-specific poly(T) eTR in a promoter-like signature roughly 5 kb upstream of *CCR6* strongly colocalizing with rheumatoid arthritis, neutrophil percent, and total protein (PP H4 = 0.996, 0.945, 0.929 respectively, *P =* 1.234e-39) (Fig. S20). CCR6 is a chemokine receptor that regulates Th17 migration^85^ and marks memory B cell precursors^86^. Individuals with rheumatoid arthritis have been found to have increased levels of B cells expressing *CCR6*, with the shift predominantly occurring in the naive B cell subset^87^. CCR6 binds to ligand CCL20, triggering an inflammatory cascade involving neutrophil recruitment^88^ and changes in acute phase protein concentrations, consistent with our colocalization findings.

Finally, we identified a CD8_TEM_-specific poly(AT) repeat overlapping with H3K36me3 signatures in the intronic region of *ITCH* (Fig. S21). This locus demonstrated robust colocalization with both colorectal cancer risk and serum alanine aminotransferase levels (PP H4 = 0.94 and 0.91, respectively; P = 7.36 × 10^−178^). *ITCH* encodes an E3 ubiquitin ligase that modulates Wnt/β-catenin signaling during colorectal cancer progression^89^. Importantly, *ITCH* expression positively correlates with CD8+ T cell tumor infiltration through its role in PD-L1 degradation, suggesting a potential mechanistic link between structural variation and immune surveillance^90^.

## Discussion

Our genome-wide analysis of TRs across 28 circulating immune cell types unveils a landscape of over 62,000 unique *cis*-acting single-cell expression quantitative trait TR loci (sc-eTRs), demonstrating the remarkable regulatory potential of repeat sequences, and contributing substantial additional signal to the systematic analyses of SNV and indel variation reported in accompanying TenK10K Phase 1 manuscripts^21^.

We conservatively identified 4,283 candidate causal sc-eTRs, which generally showed weak linkage disequilibrium (LD) with the lead SNV of their respective loci. Our work demonstrates that direct TR genotyping is essential for accurately capturing the unique contributions of this class of variation to gene expression and other traits, and that prior studies using SNV- and imputation-based approaches for association studies are likely to have missed many potentially causal TR loci.

Our findings reveal important insights into TRs as context-sensitive modulators of gene expression. We propose that some TRs can function both as continuous modulators and binary switches. For instance, at the *FRA10AC1* locus, we observed that repeat length positively associates with gene expression within the spectrum of common variation tested in our study, while larger pathogenic expansions have been reported to induce gene silencing^91^. This non-linear relationship may be more widespread, emphasizing the need to assess both the magnitude and direction of sc-eTR effects across the entire repeat length spectrum and in different cellular contexts. Indeed, some sc-eTRs achieving statistical significance across multiple cell types may appear shared but can still exert distinct, cell type-specific effects on expression.

Our work demonstrates the value of including epigenetic analysis to understand how TRs modulate gene expression. Although repeat expansions have been widely associated with gene silencing via epigenetic modifications^92,93^, our genome-wide analysis expands the catalog of cases where increasing TR length can often increase the expression of nearby genes^9^. Future epigenetic studies with larger sample sizes will help elucidate the mechanisms of cell type-specific dynamic genetic regulation.

The substantial number of novel sc-eTR associations we discovered underscores the need for directly genotyped repeat-inclusive genomic catalogs to perform downstream analysis currently out of reach, such as Mendelian randomization. Availability of these resources will also improve the power and sensitivity of existing analyses, allowing us to colocalize loci even when the fine-mapped TR is not well tagged by neighboring SNVs.

Despite computational advances, genotyping uncertainty remains a challenge for TR loci: current short-read sequencing technologies still struggle to accurately capture subsets of TR variation, including repeats found in low complexity regions (Note S1). Long-read sequencing holds promise for improving genotyping accuracy. Identifying contexts in which short-read sequencing is sufficient and those where the additional resolution of long-read sequencing is helpful will optimize the application of long-read sequencing in population-scale studies.

Our work highlights ongoing challenges in modeling TR variation, which include both stepwise mutations and repeat instability of large expansions^94^. Addressing these challenges will require continued refinement of analytical tools, such as considering both germline and somatic mutation models, as well as evaluating allele-specific effects on gene expression variation. Laboratory validation of *in silico* findings remains critical, with advances in high-throughput CRISPR screening and synthetic biology approaches^95^ offering exciting opportunities to systematically validate the functional impact of TRs in relevant cellular contexts.

In conclusion, this work shows that TR variation exerts substantial effects on cell type-specific gene expression, and that direct analysis of this class of variation is an important complement to SNV-centric approaches for deciphering genetic regulation. Future efforts toward a more comprehensive genomics approach—integrating diverse forms of genetic variation, from single nucleotides to complex structural variants, alongside epigenetic and environmental factors—will provide valuable insights into human biology, disease etiology, and therapeutic development.

## Methods

### TenK10K Phase 1 dataset processing

The TenK10K Phase 1 resource, including sample quality control, whole-genome sequencing, and single-cell RNA processing, is described in the accompanying manuscript by Cuomo et al^21^. Phase 1 comprises 1,925 individuals with matched WGS and scRNA-seq from two cohorts: the Tasmanian Ophthalmic Biobank (950 individuals of European ancestry without ocular disease) and BioHEART (975 individuals of European ancestry with diagnosed or suspected cardiac conditions).

In brief, peripheral blood samples were collected from all participants, with library preparation differing by cohort: Tasmanian Ophthalmic Biobank samples were processed using the KAPA Hyper PCR-free kit (Roche), while BioHEART samples were prepared using the DNA PCR-free sequencing library kit (Illumina). Samples underwent short-read WGS (30x) using the Illumina NovaSeq 6000 platform (2 × 150bp) and were aligned to hg38 using DRAGMAP (v1.3.1), as per DRAGEN-GATK best practices. To generate scRNA-seq data, peripheral blood mononuclear cells were isolated from each sample before undergoing single-cell RNA capture and barcoding (10x Genomics), multiplex sequencing using Illumina NovaSeq 6000 platform, alignment to genome reference (hg38, Gencode release 44, Ensembl 110) using STAR^96^, and demultiplexing with vireo^97^ (v2.1.0) in the Demuxafy implementation. Poor genotyping and/or sample quality, cryptic relatedness, and ancestral outliers were removed, resulting in 1,925 individuals with paired WGS and scRNA-seq data.

This study complied with all relevant ethical regulations and was overseen by the Human Research Ethics Committees at the Royal Children’s Hospital (Melbourne, Australia), Northern Sydney Local Health District (Sydney, Australia), St. Vincent’s Hospital (Sydney, Australia), and University of Tasmania (Hobart, Australia). Informed consent was obtained from all participants.

### TR catalog curation

A catalog of 4,918,794 loci was generated by merging several existing catalogs:

disease-associated loci (72 loci)^98^, v17 hg38 reference file from GangSTR (1,340,266 loci)^23^, hg38 reference file from HipSTR (1,634,957 loci)^24^, Illumina’s polymorphic catalog based on the 1000 Genomes Project (174,293 loci)^26^, hg38 reference file from TRGT (171,146 loci)^29^, polymorphic catalog of TR loci based on 51 haplotype-resolved assemblies from the Human Pangenome Reference Consortium (1,573,403 loci)^27,29^, and a catalog generated by Tandem Repeat Finder (TRF)^22^ (v4.09) consisting of TR loci in hg38 that span at least 9bp. The TRF catalog was generated using the following settings: match = 2, mismatch = 7, indel penalty = 1000000, PM = 80, PI = 10, minscore = 8, and maxperiod = 2000.

Merging of the TR catalogs is fully described in a separate manuscript by Weisburd and Dolzhenko et al.^28^. Briefly, component catalogs were parsed sequentially, and additional loci were added to the final catalog unless their coordinates overlapped existing included loci by 66% or more and had the same motif. Finally, to improve local read alignment for TR loci with nearby repeats, locus definitions in the catalog were modified to include adjacent repeats, if any, resulting in compound locus definitions (locus definitions with more than one TR variant specified). Compound locus definitions include a maximum of 3 TRs which are separated from each other by no more than 6bp.

### Polymorphic TR catalog curation

TR loci must be polymorphic in order for an association to exist between changes in repeat length and gene expression. To identify polymorphic TR loci in the cohort, ExpansionHunter (v5)^32^ (threads = 16, analysis-mode = streaming) was run using the aforementioned genome-wide catalog on 200 randomly selected ‘XX’ individuals.

Using Hail Query v0.2.126 (https://github.com/hail-is/hail), we removed chrY, chrM, and monomorphic loci from the resulting VCF, producing a polymorphic catalog of 2,638,529 loci (Supplementary Files).

### Benchmarking against Simons Genome Diversity Project (SGDP) samples

The performance of ExpansionHunter (v5)^32^, GangSTR (v2.5)^23^, and HipSTR (v0.6.2)^24^ were benchmarked using capillary electrophoresis results of hypervariable microsatellite loci. Specifically, WGS data and capillary electrophoresis results of 120 samples from the Simons Genome Diversity Project (SGDP) were downloaded from publicly accessible websites^34^. FASTQ files were downloaded from ENA Archive (accession no. ERP010710) and aligned to hg38 reference genome with DRAGMAP (https://github.com/Illumina/DRAGMAP), according to National Approach to Genomic Information Management (NAGIM) guidelines (https://queenslandgenomics.org/qldgenomics-updated/wp-content/uploads/2021/02/NAGIM-Blueprint-v20201010-Final-v1.2.2.pdf). Capillary electrophoresis results (in bp) corresponding to 627 STR loci were downloaded from (https://web.stanford.edu/group/rosenberglab/data/pembertonEtAl2009/combinedmicrosats_627loci_1048indivs_numRpts.stru). Primer sequences and the repeat motif of each target PCR amplicon were downloaded from (https://web.stanford.edu/group/rosenberglab/data/pembertonEtAl2009/Pemberton_AdditionalFile1_11242009.txt). We filtered for motif structures that were simple (consisting of one motif) and pure (without any sequence interruptions), resulting in a subset of 482 loci. Primer sequences of the selected loci were then mapped to hg38 reference genome using *in silico* PCR^99^. 94 loci were removed from the dataset as primer sequences could not be mapped to hg38. This produced a PCR validation dataset of 388 STR loci.

To confirm the TR motif, the number of repeat copies found in the reference genome, and the coordinates of each TR locus, TandemRepeatFinder^22^ was run on each target PCR amplicon using the settings match = 2, mismatch = 30, delta = 30, PM = 80, PI = 10, minscore = 16, maxperiod = 2000. TR loci initially defined on the antisense strand were further processed by taking the reverse complement of the sequence and re-performing TandemRepeatFinder to standardize all TR locus definitions by the sense strand.

To calculate the repeat copy allele from the PCR base pair result, the following formula was used at each TR locus: c = r + (w - l)/s, where *c* is the repeat copy, *r* is the number of repeats in hg38, *w* is the PCR amplified fragment (bp), *l* is the length of the PCR amplicon (bp) in hg38, and *s* is the motif length (bp) of the target TR^100^. Repeat copy alleles were rounded down to the nearest whole number. PCR genotypes were masked for a sample at a particular locus if an indel was identified within the PCR target region but outside of the TR locus. Indels were identified using publicly accessible FermiKit calls (https://github.com/lh3/sgdp-fermi).

Using the coordinates of each TR locus, hg38 variant catalogs were created for ExpansionHunter, GangSTR, and HipSTR. All three callers were used to genotype 120 WGS samples. ExpansionHunter was run with --analysis-mode = streaming and --threads = 16 options. GangSTR was run using default parameters. HipSTR was run using the joint calling option.

In a subset of loci, non-zero offsets were applied to correct the PCR-derived repeat copy alleles. We followed a previously published protocol where, at each locus, every discordant call made by a TR caller relative to the PCR-derived genotype was recorded and the magnitude of the discordance was scored^101^. The offset was scored as a 1 if applying the offset to the genotype produced by the TR caller recovered both PCR-derived alleles. A score of 0.5 was given if the genotype produced by the TR caller was homozygous but applying the offset recovered only one PCR-derived allele. Lastly, a score of 0.25 was given if the genotype produced by the TR caller was heterozygous but the offset recovered only one PCR-derived allele. At each locus, offset calculations were calculated separately for each of the three TR callers. Only loci with at least 20 calls were considered. An offset was applied to the PCR-derived genotype only if it was the highest-ranking offset in calls made by all three TR callers at a particular locus.

### Validation with long-read sequencing

#### HiFi sequencing methods

Cells frozen in 90% FBS:10% DMSO were defrosted at room temperature. The cells were pelleted at 4°C, 500g for 5 minutes, washed with 100 µL of 1x PBS, and then pelleted again under the same conditions. The supernatant was discarded, and the pellet was processed immediately using the Nanobind CBB DNA extraction kit (102-301-900, PacBio). Cell counts ranged from 1.6 to 4.22 × 10^6^ cells, and extracted DNA was eluted in 150 µL of EB buffer.

DNA samples were homogenized using a Diagenode Megaruptor with the 3 DNAFluid+ Kit (E07020001, Diagenode) under the following conditions: volume 150 µL, speed 40, and concentration 50 ng/µL. Subsequently, 3 µg of material was diluted in low TE to a final volume of 130 µL. Shearing was performed with the Megaruptor shearing kit (E07010003, Diagenode) at speed 30 or 31, aiming for average fragment lengths of 15–24 kb. Clean-up and concentration of the sheared material were conducted using SMRTbell clean-up beads (102-158-300, PacBio), and the DNA was eluted in 47 µL. Average fragment lengths were determined using the Femto Pulse system with the Genomic DNA 165kb Analysis Kit (FP-1002–0275, Agilent).

SMRTbell libraries were prepared using the SMRTbell^®^ Prep Kit 3.0 (102-141-700, PacBio) following standard procedures, including unique barcoding of each sample with the SMRTbell Adapter Index Plate 96A (102-009-200, PacBio). Size selection was performed with AMPure PB beads (102-182-500, PacBio) at a 2.9x ratio. Final library sizes were confirmed with the Femto Pulse, and libraries were diluted to below 60 ng/µL before the ABC loading procedure. SMRT library fragment lengths ranged between 12.895 and 26.001 kb. Sequencing was performed using the following PacBio products: Revio sequencing plate (02-587-400), Revio Polymerase kit (102-739-100), and Revio SMRT cell tray (102-202-200). On-plate loading concentration was set to 250 pM, with 30-hour movie times. SMRT libraries that did not yield 90 Gb using one SMRT cell were pooled for additional sequencing in “top-up” runs.

#### Alignment & TR genotyping

The resulting uBAM files were aligned to the hg38 reference genome using minimap2 (v2.28-r1209) with the following parameters: -y --secondary=no --MD -a -x map-hifi. Tandem repeats were genotyped in individual samples using TRGT (v1.1.0) with a custom catalog of polymorphic tandem repeats. The resulting tandem repeat genotypes were then merged into a single VCF for downstream analysis, also using TRGT.

### TR genotyping and filtering

ExpansionHunter (v5)^32^ was used to genotype TRs defined in the polymorphic TR catalog, producing diploid TR genotypes (in repeat lengths) for each sample using realigned reads. VCFs were merged using mergeSTR (TRTools v5.0.2)^102^ and filtered to exclude loci meeting the following criteria: locus-level call rate < 90%, observed heterozygosity < 0.00995 (corresponds to a non-major allele frequency < 0.5%), or locus-level Hardy-Weinberg equilibrium (binomial test, *P* < 1×10^−6^). Calls that were more than 30 repeats less or 20 repeats greater than the mode allele at a particular locus were set to missing. This threshold was determined based on comparisons with a publicly available truth set^27^ to assess genotyping accuracy. All samples had a sample-level TR call rate greater than 99%.

### SNV genotyping and filtering

Calling of SNPs and indels was then performed using GATK4 HaplotypeCaller in DRAGEN mode. We removed monomorphic and rare variants, defined as having a minor allele frequency < 1%. Multiallelic variants were split into biallelic variants. Detailed sample and variant (SNV) QC is described in the accompanying manuscript by Cuomo et al^21^.

### eTR and eSNV association analysis

To study the effect of TR repeat length variation on gene expression in a cell-type specific context, we performed sc-eTR discovery with associaTR (TRTools v5.0.2)^102^, a TR-aware association testing framework that performs length-based tests for multiallelic TR loci against a continuous phenotype.

Prior to sc-eTR discovery, scRNA-seq data was processed using approaches described in detail in the accompanying manuscript by Cuomo et al^21^. Briefly, we aligned and quantified scRNA-seq reads using cellranger (v7.2.0) (10X Genomics). We removed cells that were unassigned to individuals or classified as doublets by at least two out of the three methods: scds^103^, vireo^104^, and scDblFinder^105^. We also removed cells with over 20% mitochondrial reads, cells with fewer than 1,000 or greater than 10,000 expressed genes, and cells with less than 800 reads detected to exclude potential doublets and low-quality cells. Cell type annotation was performed using the hierarchical scPred^38^ method, using a published CITE-seq dataset^106^ to train the model.

scRNA transcript counts were then further processed to generate pseudobulk samples. In detail, single-cell-level normalization was performed using scanpy^107^ (v1.9.3) - raw transcript counts were normalized for sequencing depth, log-transformed, and corrected for batch effects - prior to mean aggregation at the sample level. This workflow aligned with the recommendations by Cuomo et al.^108^ for optimizing pseudobulk eQTL mapping workflows.

Subsequently, lowly expressed genes (expressed in fewer than 1% of cells) were excluded from each cell type. Expression values for remaining genes were quantile-normalized to a standard normal distribution prior to eQTL mapping.

Expression values were adjusted separately for each cell type to control for karyotypic sex, age, population structure, and technical variation in expression. For population structure, we selected the top 12 principal components (PCs) produced from the principal component analysis of SNV genotypes from all European samples that passed whole genome sequencing quality control.

To control for technical variation in gene expression, we used the top 6 PCs produced from the principal component analysis of mean aggregated normalized RNA transcript counts per cell type. We tested the effect of 1–10 principal components sequentially by adding them one at a time into a linear regression model of CD8_TEM_ *cis* sc-eTR analysis and identified that a model consisting of 6 PCs identified the greatest number of eGenes (Fig. S22).

For each TR within 100 kb of a gene, we used associaTR (TRTools v5.0.2) to perform ordinary least squares regression between the summed TR repeat length (across both alleles for each individual) and normalized gene expression, while adjusting for known confounders:

y = Wα + gβ + ϵ

where 𝑦 denotes normalized gene expression values, 𝑊 denotes the aforementioned covariates, β denotes the effect size the model is estimating, 𝑔 denotes the summed TR repeat length, and ϵ is the normally distributed error term. We used the same model for association testing of SNVs and indels within 100kb of each gene, with 𝑔 denoting the dosage of the non-reference allele.

### Meta-analysis

Meta-analysis was performed using a random effects (Dersimonian and Laird) model using the meta package in R (v4.0.0)^109^. To assess potential inflation of Type 1 error, we performed a permutation of the sample identifiers in the CD4_TCM_ subtype and repeated the regression analysis and meta-analysis. Type 1 error was well controlled (Fig. 2a).

### Multiple testing correction

Gene level p-values were computed by combining variant-level p-values of variants using the ACAT-V test^110^ based on the Cauchy combination. We identified a set of top sc-eTRs by selecting the eTR with the lowest nominal p-value for each gene in each cell type. We then corrected for multiple testing across all genes tested, for every cell type, using the Storey q-value procedure^111^, reporting results at FDR < 5%. Original p-values accompany case examples discussed in the main text of the manuscript, with the minimum original p-value provided if the sc-eTR was shared across multiple cell types.

### Multivariate adaptive shrinkage (mash) analysis

To account for global effect size sharing and power differences across cell types, we ran mashR (v0.2.79)^36^, a multivariate adaptive shrinkage method, following the documentation for eQTL analysis (https://stephenslab.github.io/mashr/articles/eQTL_outline.html). Briefly, we obtained the effect sizes and standard errors for every sc-eQTL (FDR <5%) that was tested in every cell type. We used the sc-eQTLs to compute the ‘data-driven’ covariance matrix using an extreme deconvolution method involving the top five principal components. The ‘data-driven’ and ‘canonical’ covariance matrices were then used to fit the mash model to all chr22 variants tested in all cell types (‘random’ tests). Finally, the mash model was used to calculate the posterior effect sizes of sc-eQTLs across all cell types. We performed this analysis for sc-eSNVs and sc-eTRs separately.

### Cell type specificity analysis

Cell type specificity of sc-eTRs was assessed using a pairwise meta-analysis approach. For each significant sc-eTR (FDR < 5%), we conducted pairwise meta-analyses comparing its effect size in the original cell type to its effect sizes in each of the other cell types.

Meta-analysis was performed using a random effects (Dersimonian and Laird) model with the meta package in R (v4.0.0)^109^. We compared the resulting meta-analysis p-value to the original p-value obtained from the single-cell eQTL analysis. If the meta-analysis p-value was lower than the original p-value, we concluded that the sc-eTR was shared between the two cell types.

To determine cell type specificity, we examined the results of all pairwise comparisons for each sc-eTR. An sc-eTR was considered specific to one cell type (i.e. cell type-specific) if the meta-analysis p-value was consistently higher than the original p-value across all cell types where the gene was expressed in at least 1% of cells.

### Fine-mapping and conditional analysis

Prior to fine-mapping, we identified and removed indels that represent TR alleles, similar to Margoliash et al^11^. We excluded indels that were 1) found in TR intervals (as defined in our catalog) and 2) composed of whole copies of the TR motif, including its cyclical representations. Conservatively, we retained indels that were not perfect multiples of the TR motif and also retained indels where at least one base would represent a sequence impurity in the TR interval.

We fine-mapped each eGene passing multiple testing correction (FDR < 5%) using SuSiE-RSS (v0.12.35)^112^. The fine-mapping region for each gene was defined as the window extending 100 kilobases (kb) upstream of the transcription start site (TSS) and 100 kb downstream of the transcription end site (TES). We used the summary association statistics derived from meta-analysis and restricted SuSiE-RSS to identify a maximum of one causal credible set in all runs. We used SuSIE’s posterior inclusion probability (PIP) with non-default parameter prune_by_cs set to TRUE, as per Margoliash et al^11^. We conservatively considered an sc-eTR to be candidate causal if it was the strongest signal in the eGene and had a SuSIE PIP of at least 0.7. We then performed conditional analysis on every candidate causal sc-eTR by performing association testing as previously described, conditioned on the summed repeat dosages of the candidate causal sc-eTR.

### Functional and genomic annotation of eTRs

We used publicly accessible resources to annotate the sc-eTRs, including ENCODE (v3) *cis* candidate regulatory elements (cCREs) combined from all cell types (https://hgdownload.soe.ucsc.edu/gbdb/hg38/encode3/ccre/encodeCcreCombined.bb), MANE (v1.0) (https://ftp.ncbi.nlm.nih.gov/refseq/MANE/MANE_human/), and TR PhEWAS hits^14^ (Table S3).

### Methylation analysis

We used pb-CpG-tools (v2.3.2) to obtain site methylation probabilities for all CpG sites genome-wide in 25 PacBio HiFi sequencing samples, corresponding to long-read sequencing of 25 individuals in Phase 1 of TenK10K.

At each CpG site, methylation probabilities were quantile-normalized to a standard normal distribution, and CpG sites were retained for eQTL mapping if the standard deviation of methylation values was greater than 0.02, as per Trujillo et al.^10^

For each candidate causal TR within 5 kb of a CpG site, we used associaTR (TRTools v5.0.2)^102^ to perform ordinary least squares regression between the summed TR repeat length (across both alleles for each individual) and normalized methylation probability, while adjusting for known confounders:

y = Wα + gβ + ϵ

where 𝑦 denotes normalized gene expression values, 𝑊 denotes the aforementioned covariates (karyotypic sex, age, population structure), β denotes the effect size the model is estimating, 𝑔 denotes the summed TR repeat length, and ϵ is the normally distributed error term.

Bonferroni correction was applied to adjust for the number of TR-CpG pairs tested, with significant associations reported with a Bonferroni-adjusted p-value < 0.05.

### Colocalization

We downloaded publicly accessible SNV GWAS summary statistics for inflammatory bowel disease^59^ (https://www.dropbox.com/scl/fi/9yyfkpa09x2bm3s56q1t2/liu-2022-east-asian-gwas.tar.gz?rlkey=96ezojmceei0ofe71z3pe7gm3&dl=0), systemic lupus erythematosus^61^ (https://www.ebi.ac.uk/gwas/studies/GCST003156), rheumatoid arthritis^60^ (https://www.ebi.ac.uk/gwas/studies/GCST90132223), COVID-19^69^ (https://www.ebi.ac.uk/gwas/studies/GCST011071), lung cancer^65^ (https://www.ebi.ac.uk/gwas/studies/GCST004748), breast cancer^63^ (https://www.ebi.ac.uk/gwas/studies/GCST004988), Parkinson’s disease^71^ (https://www.ebi.ac.uk/gwas/studies/GCST009325), malignant lymphoma^66^ (https://www.ebi.ac.uk/gwas/studies/GCST90018878), prostate cancer^67^ (https://www.ebi.ac.uk/gwas/studies/GCST90274713), Alzheimer’s disease^70^ (https://www.ebi.ac.uk/gwas/studies/GCST90027158), colorectal cancer^64^ (https://www.ebi.ac.uk/gwas/studies/GCST90129505), IgA nephropathy^62^, and lymphocytic leukemia^68^ (https://www.ebi.ac.uk/gwas/studies/GCST90011814).

We downloaded SNV and imputed STR UKBB GWAS summary statistics for 44 blood cell count and serum traits^11^ from (https://gymreklab.com/science/2023/09/08/Margoliash-et-al-paper.html). Detailed SNV and STR methylation eQTL summary statistics^10^ were provided by Alejandro Martin-Trujillo upon request.

Colocalization was performed using coloc.abf function from coloc (v5.2.3)^72^. SNV-only colocalization was performed using SNV GWAS summary statistics and sc-eSNVs in our dataset with eQTL type set to ‘quant’ and sdY = 1 as expression was quantile normalized to a standard normal distribution. Prior to TR and SNV joint colocalization using either 1) imputed STR and SNV UKBB GWAS summary statistics or 2) STR and SNV methylation eQTL summary statistics, we harmonized the TR locus definitions in the respective catalogs with those in our TR catalog by verifying, for each pair of intersecting locus definitions, whether one motif was the reverse complement and/or cyclical shift of the other. Colocalization with SNV and STR methylation eQTL summary statistics was performed separately for each distinct methylation probe reported in Trujillo et al.^10^.

We hypothesized that candidate causal sc-eTRs could drive GWAS signals if the following conditions were met: (1) strong colocalization evidence indicating that the trait and gene expression are driven by the same variant (PP H4 ≥ 0.8), (2) evidence that the sc-eTR is the lead variant driving changes in gene expression and is confidently fine-mapped (PIP ≥ 0.7 using a single causal variant model), and (3) evidence that the sc-eTR is in at least moderate linkage disequilibrium (LD, r^2^ ≥ 0.5) with a variant cataloged in the GWAS window.

Linkage disequilibrium was calculated by taking the square of the Pearson correlation of the genotypes of every pair of variants (summed repeat dosage for TRs or genotype dosage of the non-reference allele for SNVs).

## Supplementary Material

Supplement 1

Supplement 2

Supplement 3

## Figures and Tables

**Figure 5:**
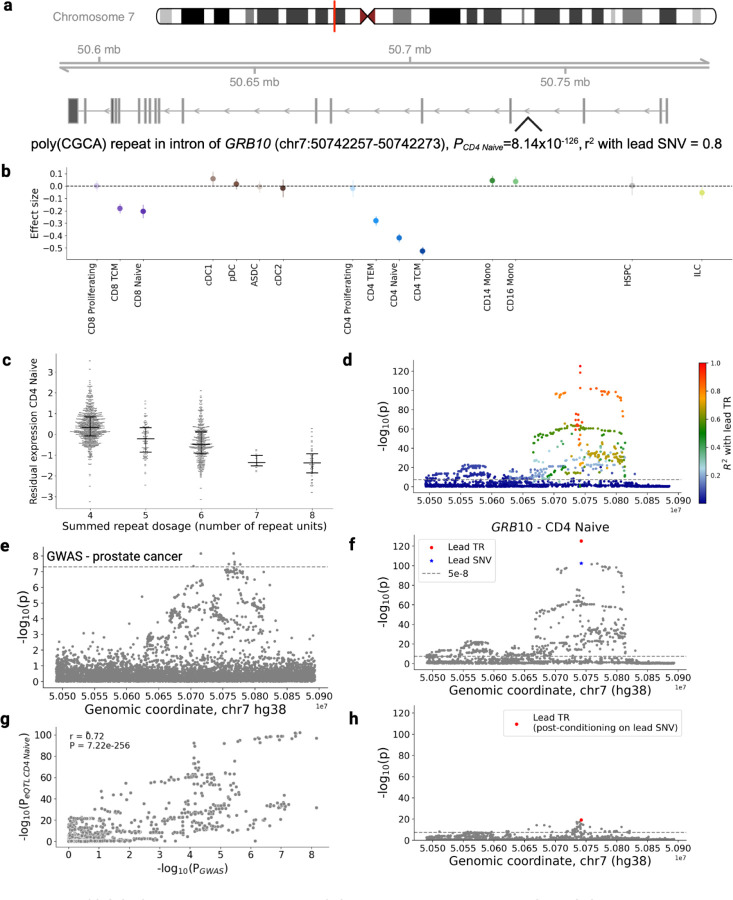
poly(CGCA) repeat in the intron of *GRB10* colocalizes with GWAS for prostate cancer. **a**, Locus zoom plot of *GRB10* using Gviz^82^, loaded with GENCODE v44 track. **b**, Distribution of effect size of the sc-eTR in immune cell types which had sufficient gene expression for association testing (Methods). Error bars correspond to 95% confidence intervals. **c**, Association between repeat length and residual gene expression in CD4+ naive T cells. **d**, sc-eQTL association signals for the candidate causal sc-eTR and other SNVs and TRs within +/− 100kb of the gene body, colored by strength of linkage disequilibrium with the candidate causal TR (R^2^). **e,** Prostate cancer GWAS association signals for the equivalent window (+/− 100kB of the gene body of *GRB10*). **f**, Association signals for the candidate causal sc-eTR (red circle), the lead eSNV (blue star), and other SNVs and TRs within +/− 100kb of the gene body (gray points). **g**, Association between p-values of overlapping variants in the CD4+ naive T cell sc-eQTL dataset and the prostate cancer GWAS catalog. **h,** Association signals for SNVs and TRs within +/− 100 kb of the gene body, conditioning each on the genotype of the lead eSNV.

## Data Availability

The polymorphic variant catalog is provided in the supplement. Summary level association statistics are provided as supplementary files. Raw summary association statistics will be available at 10.5281/zenodo.15009519.
