# Supplementary material for: Polymorphic tandem repeats influence cell type-specific gene expression across the human immune landscape
Source: bioRxiv. 2025 Apr 9:2024.11.02.621562. Preprint. [Version 2] doi: 10.1101/2024.11.02.621562 (PMC12026411; doi:10.1101/2024.11.02.621562)
Supplement: Supplement 3 [file media-3.pdf]

Supplementary Information

Polymorphic tandem repeats influence cell type-specific gene expression across the human immune landscape

Supplementary Notes……………………………………………………..………………3

Supplementary Note 1: Concordance between long and short-read TR genotypes
Supplementary Note 2: Gene ontology enrichment analysis of cell type-specific eGenes
Supplementary Note 3: Fine-mapping instability assuming multiple causal variants
Supplementary Note 4: Cell type-specific associations of eTRs and expression of *GLS* and *PPP2R2B*

Supplementary Figures………………………………………………………………………4

Supplementary Figure 1: Accuracy of ExpansionHunter, HipSTR, and GangSTR as a function of sensitivity
Supplementary Figure 2: Concordance between long-read TRGT calls and short-read ExpansionHunter calls
Supplementary Figure 3: Principal component analysis (PCA) plot of TR genotypes
Supplementary Figure 4: TR eQTL (eTR) discovery in TenK10K Phase 1 (FDR < 5%) compared to two publicly available bulk eTR catalogs
Supplementary Figure 5: single-cell eTR meta-analysis discovers more unique sc-eTRs (FDR <5%) than individual cohorts
Supplementary Figure 6: Heatmap of pairwise correlations of *cis* sc-eSNV posterior effect sizes across cell types, adjusted for global effect size sharing and power differences with multivariate adaptive shrinkage
Supplementary Figure 7: Log_2(fold enrichment) of cell type-specific sc-eTRs relative to shared sc-eTRs, by MANE and ENCODE cCRE annotation
Supplementary Figure 8: poly(TAT) intronic repeat associated with *PREX1* expression in CD4+ naive T cells and CD16+ monocytes
Supplementary Figure 9: Linkage disequilibrium (LD, calculated as R^2) of lead variant with next most significant neighboring SNV in the $CD4_{TCM}$ cell type, grouped by loci where the lead variant is a SNV or a TR
Supplementary Figure 10: Distribution of the ratio of p-values of the lead variant relative to that of the next significant neighboring variant in the $CD4_{TCM}$ cell type, grouped by loci where the lead variant is a SNV or a TR
Supplementary Figure 11: Enrichment of sc-eTRs and candidate causal sc-eTRs relative to all TRs genotyped
Supplementary Figure 12: Number of eGenes (associated with fine-mapped sc-eTRs [PIP ≥ 0.7]) by loss-of-function observed/expected upper bound fraction (LOEUF) score decile
Supplementary Figure 13: Enrichment of candidate causal sc-eTRs relative to all TRs genotyped in primate-specific and Zoonomia annotations
Supplementary Figure 14: Cell type-specificity of sc-eTRs significantly associating with methylation, as measured with PacBio long-read sequencing (Bonferroni-adjusted $P < 0.05$) and significant colocalization (PP H4 ≥ 0.8) with methylation signals derived from a published dataset of SNVs and STRs
Supplementary Figure 15: Distribution of linkage disequilibrium (LD) with the lead SNV of the respective *cis* window for all sc-eTRs and for candidate causal sc-eTRs
Supplementary Figure 16: Barplot of the number of candidate causal sc-eTRs intersecting significant TR UKBB PheWAS hits, colored by gene annotation.

Supplementary Figure 17: Barplot of the number of colocalized genes (PPH4 ≥0.8) containing a candidate causal sc-eTR, per GWAS catalog. Phenotypes marked with † were colocalized using only SNVs while unmarked phenotypes were colocalized using SNVs and imputed TRs.
Supplementary Figure 18: poly(T) repeat in the promoter of *KIF16B* colocalizes with GWAS for mean platelet volume
Supplementary Figure 19: poly(GGA) repeat in the CDS of *ALMS1* colocalizes with GWAS for serum IGF-1
Supplementary Figure 20: poly(T) repeat in promoter-like signature of *CCR6* colocalizes with GWAS for rheumatoid arthritis
Supplementary Figure 21: poly(AT) repeat in intronic region of *ITCH* colocalizes with GWAS for colorectal cancer
Supplementary Figure 22: Changes in the number of *cis*-eGenes detected with the addition of expression principal components
Supplementary Figure 23: Discordance between SuSIE and FINEMAP fine-mapping tools, with a maximum of 10 credible sets specified
Supplementary Figure 24: Concordance of posterior inclusion probabilities (PIPs) produced by SuSIE and FINEMAP for all TRs and SNVs with p-value < $5x10^{-8}$
Supplementary Figure 25: Cell type-specific associations of eTRs and expression of *GLS*
Supplementary Figure 26: sc-eTR associated with *PPP2R2B* expression

Supplementary Tables……………………………………………………………………………22

All supplementary tables, apart from Supplementary Table 8, are provided as supplementary files.

Supplementary Table 1: List of single-cell expression quantitative trait TR loci (sc-eTRs) (FDR < 5%)
Supplementary Table 2: List of single-cell eTRs (nominal P-value < 0.05) that overlapped with bulk eTRs previously identified in Fotsing et al. (DOI: 10.1038/s41588-019-0521-9) and Bakhtiari et al. (DOI: 10.1038/s41467-021-22206-z)
Supplementary Table 3: List of candidate causal sc-eTRs (posterior inclusion probability ≥ 0.7)
Supplementary Table 4: List of candidate causal sc-eTRs that associate with methylation of a nearby CpG site (within 5kB) (Bonferroni adjusted p-value < 0.05)
Supplementary Table 5: List of candidate causal sc-eTRs with evidence of colocalization with orthogonal methylation data [PP H4 ≥ 0.8] (Trujillo et al. [DOI:10.1101/gr.277057.122])
Supplementary Table 6: List of candidate causal sc-eTRs that associate with methylation of a nearby CpG site (within 5kB) (Bonferroni adjusted p-value < 0.05) and show evidence of colocalization with orthogonal methylation data [PP H4 ≥ 0.8] (Trujillo et al. [DOI:10.1101/gr.277057.122])
Supplementary Table 7: List of candidate causal sc-eTRs with p-value < 5×10^{-8} post-conditioning on the lead SNV
Supplementary Table 8: Candidate causal TRs intersecting known disease-associated loci
Supplementary Table 9: List of eGenes associated with candidate causal sc-eTRs intersecting with list of genes curated in PanelApp Australia (downloaded on October 15, 2024)
Supplementary Table 10: List of candidate causal sc-eTRs intersecting UKBB TR PheWAS hits (Manigbas et al. 2024 [DOI: 10.1038/s41467-024-54678-0])
Supplementary Table 11: List of colocalized GWAS loci [PP H4 ≥ 0.8] containing at least one sc-eTR that is candidate causal for gene expression

Supplementary Notes

Supplementary Note 1: **Concordance between long and short-read TR genotypes**

Comparing long-read and short-read TR genotypes, we found that poorly concordant calls were enriched in repeat loci with multiple adjacent motifs specified (odds ratio (OR) = 26.74; Fisher's two sided $P < 2.2\text{x}10^{-16}$), reflecting the known limitation of short-read TR genotyping in low complexity regions with multiple adjacent, and possibly overlapping, repeat motifs[1] (Fig. S2).

Supplementary Note 2: **Gene ontology enrichment analysis of cell type-specific eGenes**

We tested for enrichment of eGenes associated with cell type-specific sc-eTRs among Gene Ontology (GO)[2–4] terms using a background set of 14,732 eGenes associated with all sc-eTRs. We recapitulated known functional associations between cell types and their eGenes. For example, B cell-specific sc-eTRs were enriched for eGenes involved in B cell receptor signaling and humoral immune response mediated by immunoglobulin (Fisher's exact test, $P = 1.33\times10^{-4}$ and 2.27×10^{-11} respectively). Dendritic cell-specific sc-eTRs were enriched for endocytosis-related genes ($P = 1.35\times10^{-8}$).

Supplementary Note 3: **Fine-mapping instability assuming multiple causal variants**

We attempted fine-mapping with 10 credible sets using SuSiE-RSS (v0.12.35) and FINEMAP shotgun stochastic search (v1.4.2). However, the results lacked concordance between the two methods (Fig. S23-24). Fine-mapping of meta-analysis results under a multiple causal variant assumption may also be miscalibrated using existing tools[5]. Therefore, we proceeded with fine-mapping using a single causal variant assumption.

Supplementary Note 4: **Cell type-specific associations of eTRs and expression of *GLS* and *PPP2R2B***

While rare expansions of the poly(GCA) repeat in the 5' UTR of *GLS* is known to cause reduced *GLS* transcription[6], we found common variation of the repeat to be candidate causal for expression of two genes - *GLS* and lnc-NEMP2-1 (Fig. S25, Table S8). *GLS* encodes glutaminase which regulates glutamine, a key amino acid involved in immune proliferation and cytokine production[7]. Increasing TR length was found to associate with decreasing expression of *GLS* in CD4+ naive, $CD4_{TCM,}$ $CD4_{TEM}$, $CD8_{TCM}$, and B naive subtypes (minimum $P = 2.63\times10^{-38}$). Meanwhile, increasing TR length was found to associate with increasing expression of lnc-NEMP2-1 in B and MAIT subtypes (minimum $P = 8.27\times10^{-62}$).

As another example, a CTCF-binding poly(GCT) 5' UTR repeat, the same locus associated with spinocerebellar ataxia 12[8], was found to be candidate causal for *PPP2R2B* expression, with increasing repeat length associating with decreased gene expression in $CD4_{TCM}$ ($P = 1.27\times10^{-20}$, Fig. S26). *PPP2R2B* encodes B55β, which induces T cell apoptosis through IL-2 deprivation[9,10]. Hypermethylation and subsequent silencing of *PPP2R2B* has previously been associated with autoimmune inflammation due to failed T cell apoptosis[9,10]. The effect of hypermethylation is likely most prominent in T cells, where a T cell-specific DNAse I hypersensitivity signature overlaps the TR[11].

Supplementary Figures

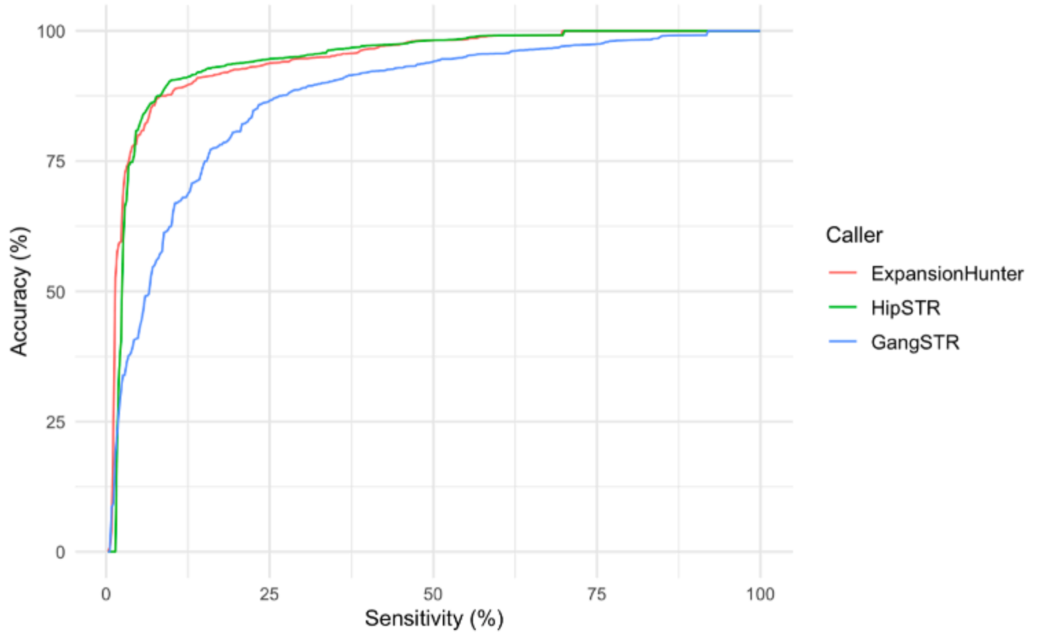

Supplementary Figure 1: Accuracy of ExpansionHunter, HipSTR, and GangSTR as a function of sensitivity. Accuracy was defined as the proportion of summed diploid genotypes that exactly match those in the PCR dataset, taking into account offsets (Methods). Sensitivity was defined as the proportion of STR loci in the catalog that was genotyped.

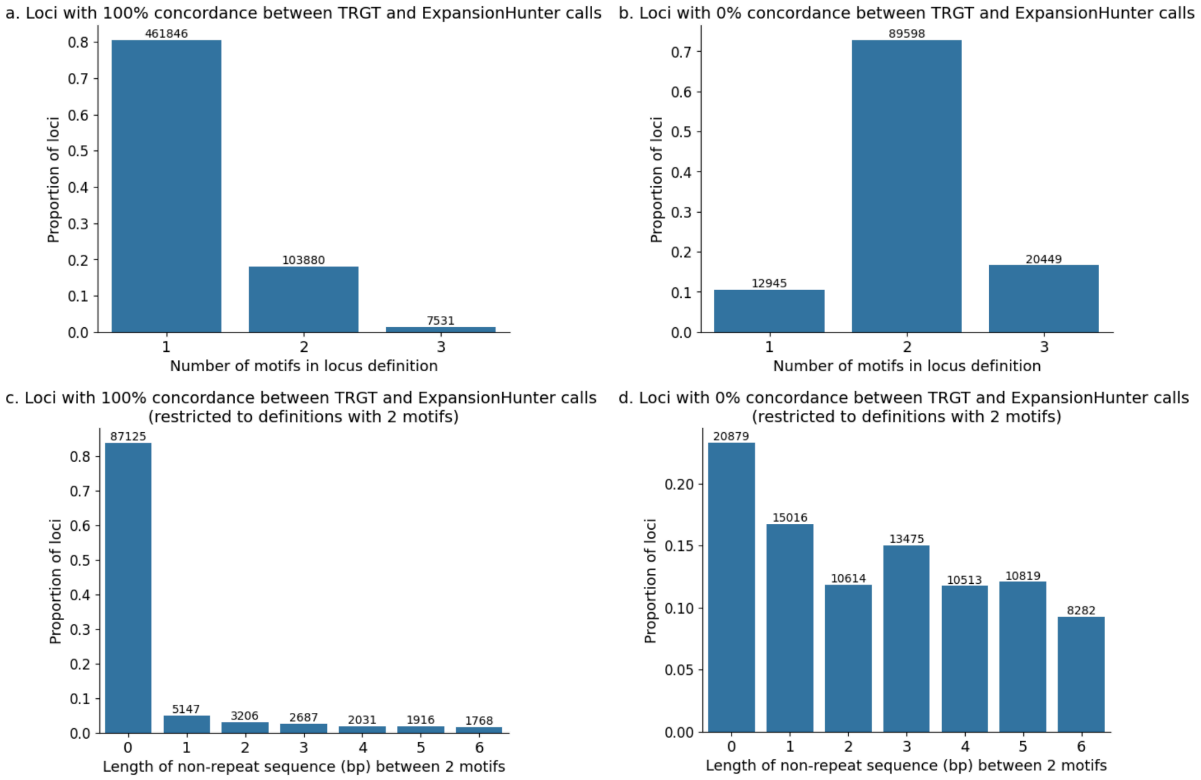

Supplementary Figure 2: **Concordance between long-read TRGT calls and short-read ExpansionHunter calls. a**, Distribution of compound motif definitions (n=25 samples) in loci with 100% concordance between long-read TRGT calls and short-read ExpansionHunter calls and **b**,

those with 0% concordance. **c,** Distribution of interrupting (non-repeat) sequence length in loci defined by 2 motifs (n=25 samples) in loci with 100% concordance between long-read TRGT calls and short-read ExpansionHunter calls and **d**, 0% concordance.

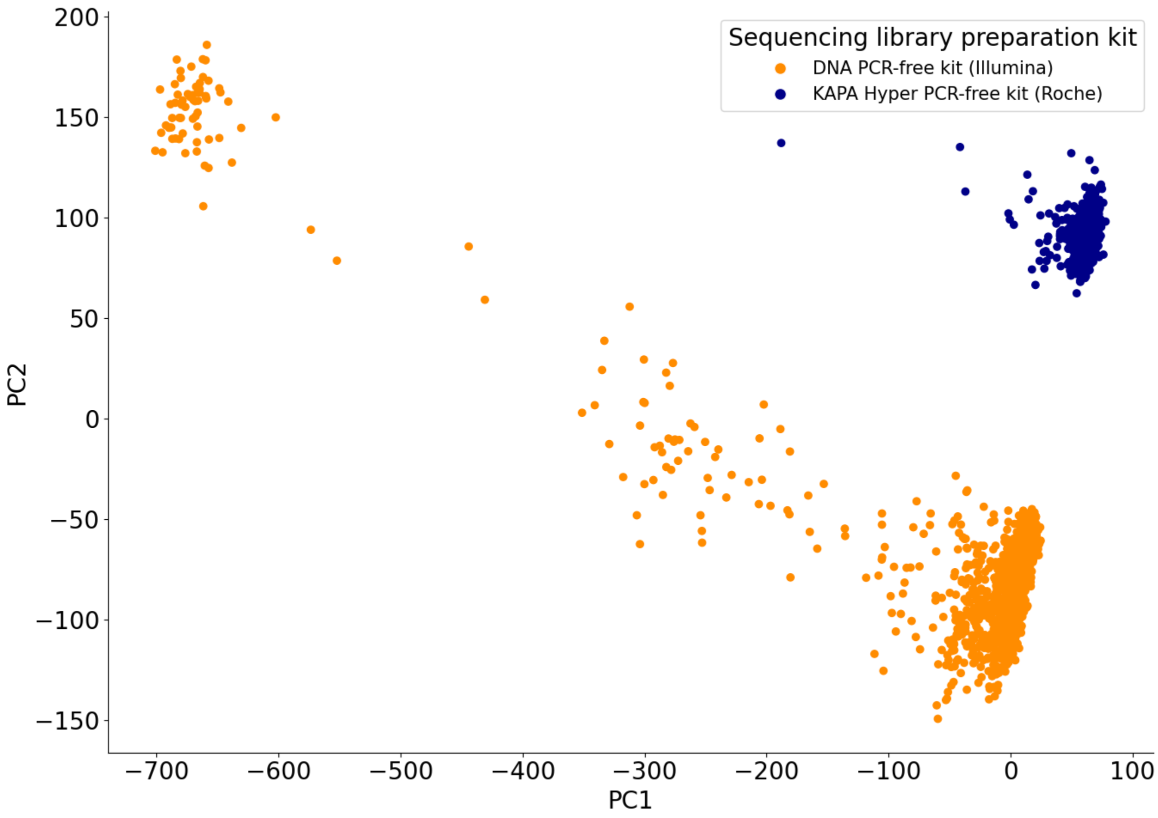

Supplementary Figure 3: Principal component analysis (PCA) plot of TR genotypes. Points are colored by sequencing library preparation kit.

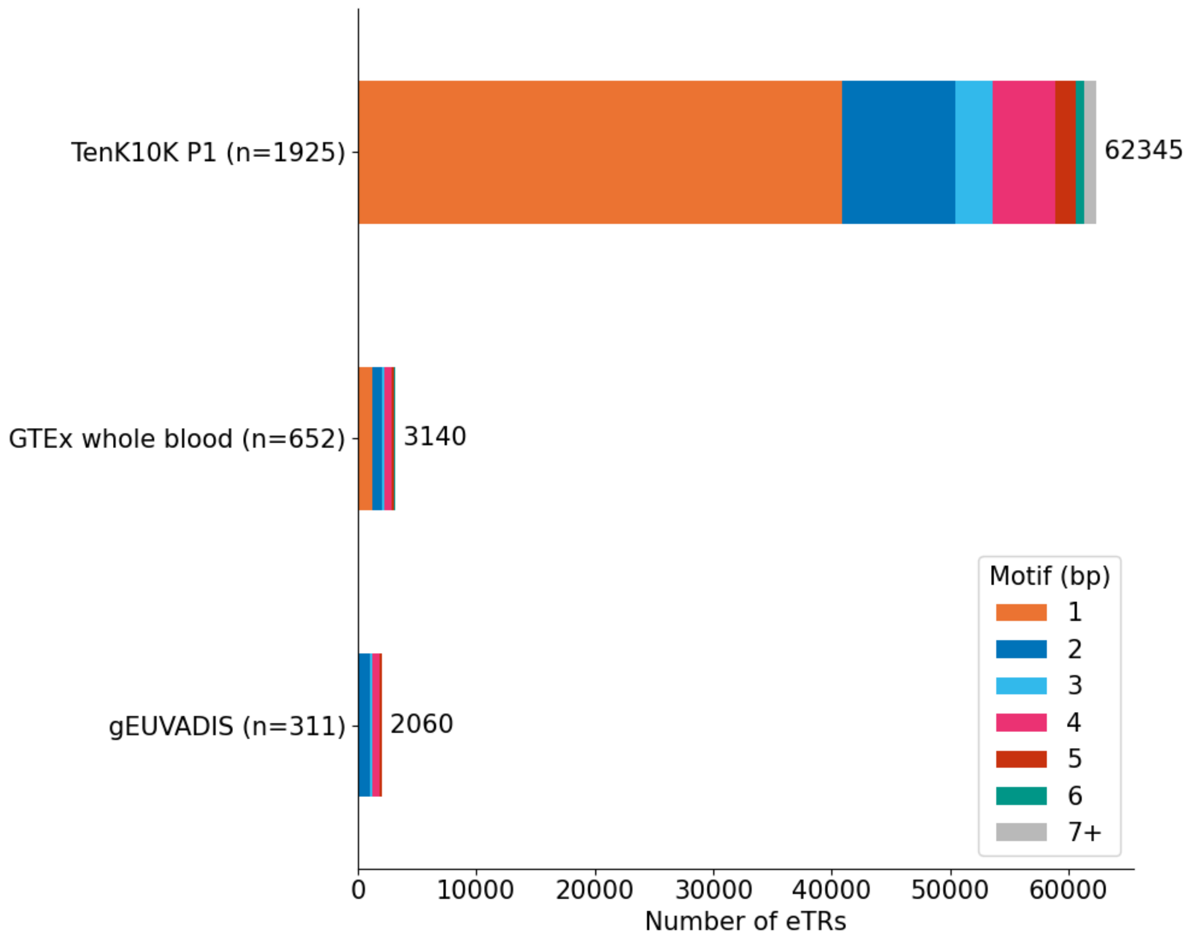

Supplementary Figure 4: TR eQTL (eTR) discovery in TenK10K Phase 1 (FDR < 5%) compared to two publicly available bulk eTR catalogs. Bulk eTR catalogs were based on GTEx[12,13] (bulk RNA-seq of whole blood; FDR < 10% for STRs and < 5% for VNTRs) and gEUVADIS[14] (bulk RNA-seq of HapMap lymphoblastoid cell lines; FDR < 5%) datasets.

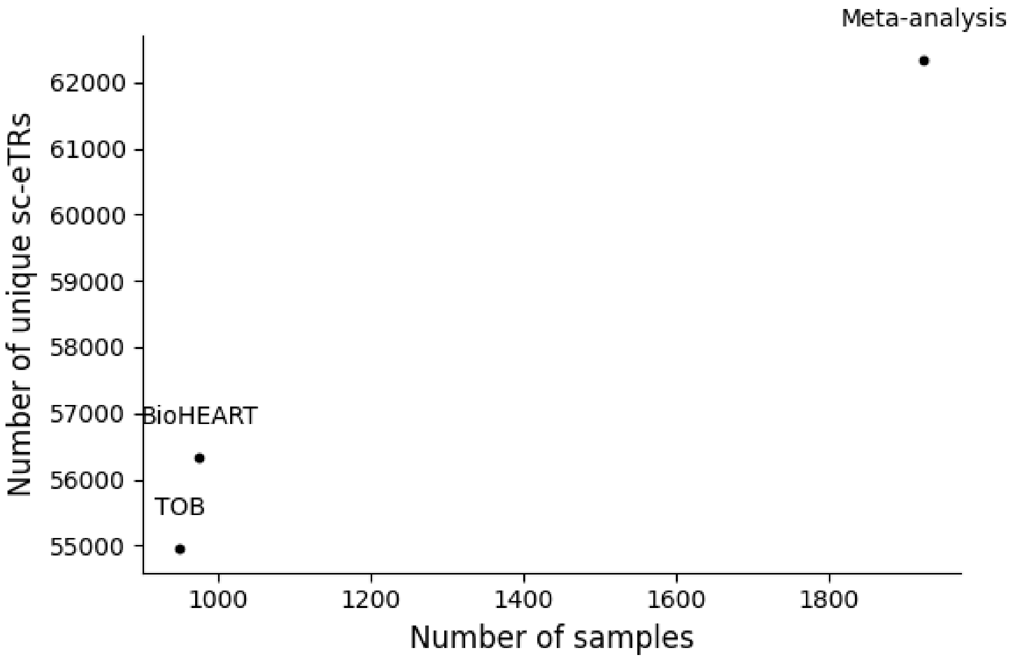

Supplementary Figure 5: single-cell eTR meta-analysis discovers more unique sc-eTRs (FDR <5%) than individual cohorts.

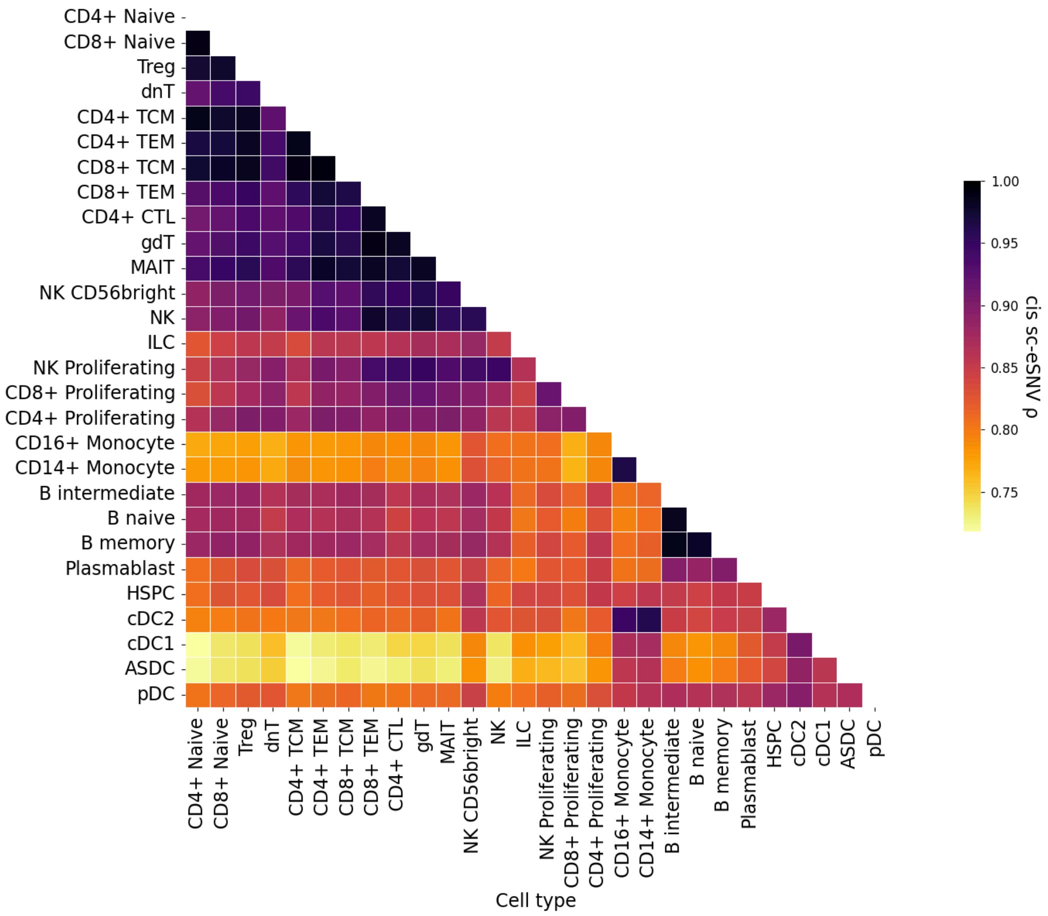

Supplementary Figure 6: Heatmap of pairwise correlations of *cis* sc-eSNV (FDR < 5%) posterior effect sizes across cell types, adjusted for global effect size sharing and power

differences with multivariate adaptive shrinkage[15]. Order of rows and columns reflect scRNA-seq cell type classification by scPred[16] (Methods).

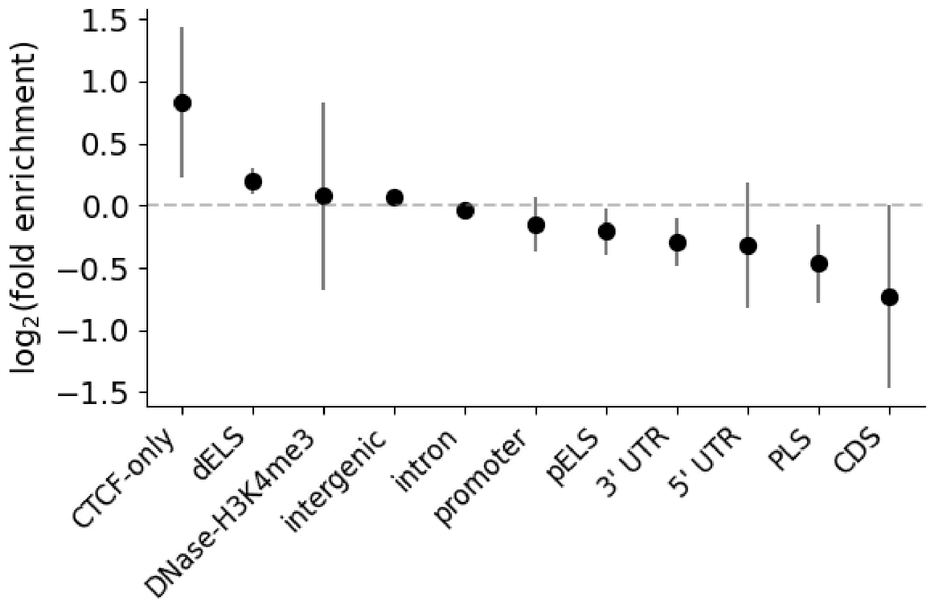

Supplementary Figure 7: **Log_2(fold enrichment) of cell type-specific sc-eTRs relative to shared sc-eTRs, by MANE and ENCODE cCRE annotation**. Error bars correspond to 95% confidence intervals. ENCODE candidate *cis* regulatory element (cCRE) annotations are abbreviated as follows: promoter-like signature [PLS], proximal enhancer-like signature [pELS], distal enhancer-like signature [dELS], CTCF binding site failing to intersecting another cCRE annotation [CTCF-only], DNase-H3K4me3 site failing to intersect promoter and enhancer signatures [DNase-H3K4me3]. MANE annotations are abbreviated as follows: 5' untranslated region [5' UTR], 3' untranslated region [3' UTR], coding sequence [CDS].

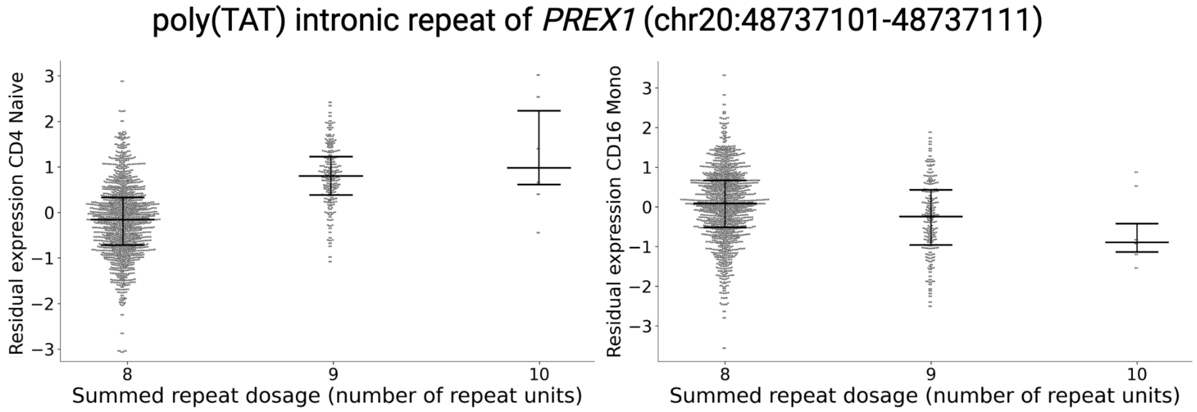

Supplementary Figure 8: **poly(TAT) intronic repeat associated with *PREX1* expression in CD4+ naive T cells and CD16+ monocytes.**

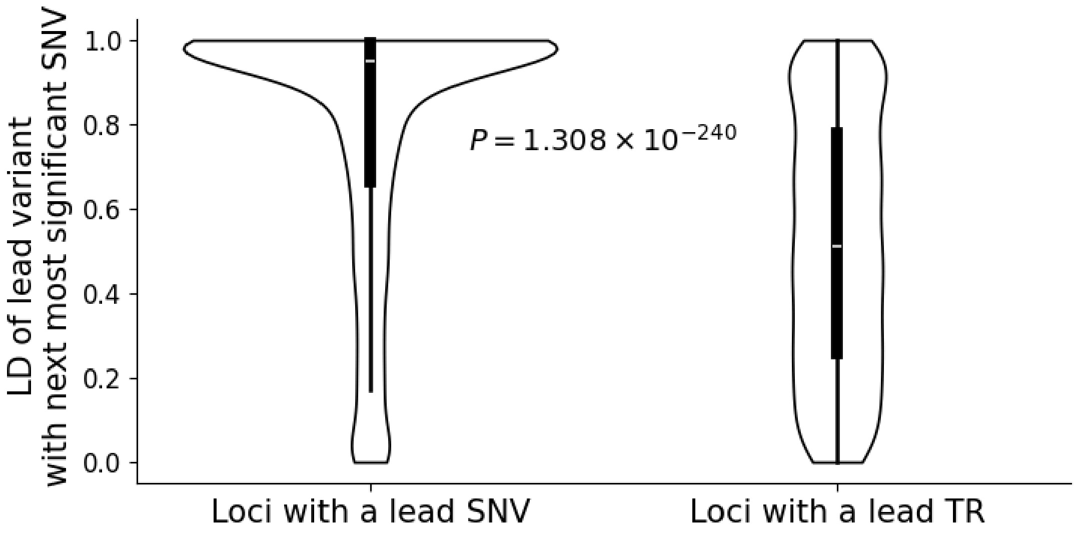

Supplementary Figure 9: Linkage disequilibrium (LD, calculated as R^2) of lead variant with next most significant neighboring SNV (within +/- 100 kb of the gene body) in the $CD4_{TCM}$ cell type, grouped by loci where the lead variant is a SNV or a TR. Lead variants have nominal $P < 5\times10^{-8}$.

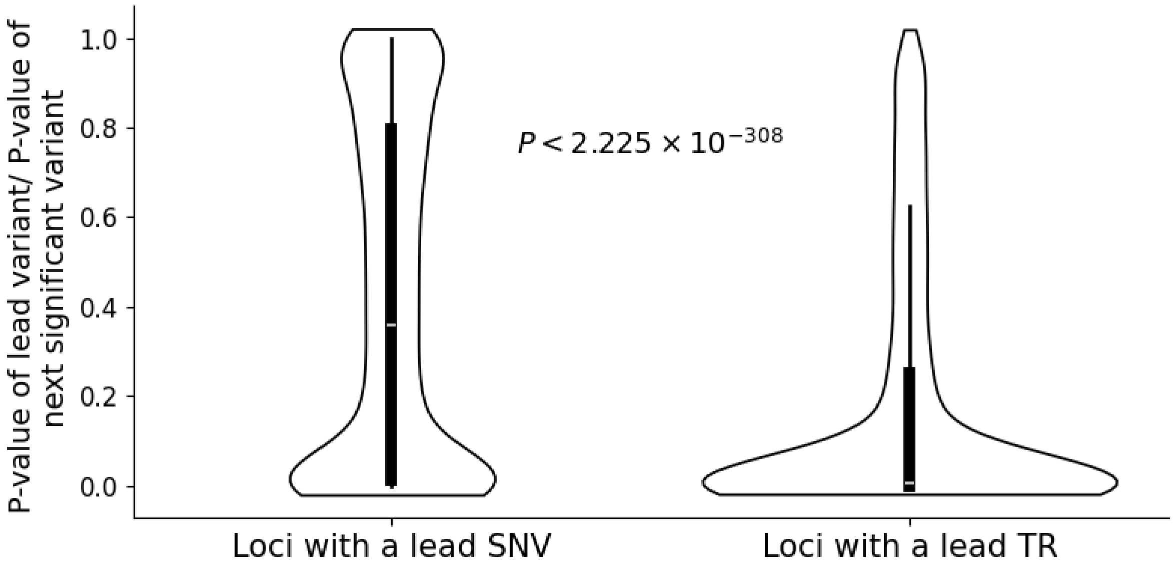

Supplementary Figure 10: Distribution of the ratio of p-values of the lead variant relative to that of the next significant neighboring variant (within +/- 100 kb of the gene body) in the $CD4_{TCM}$ cell type, grouped by loci where the lead variant is a SNV or a TR. Lead variants have nominal $P < 5\times10^{-8}$.

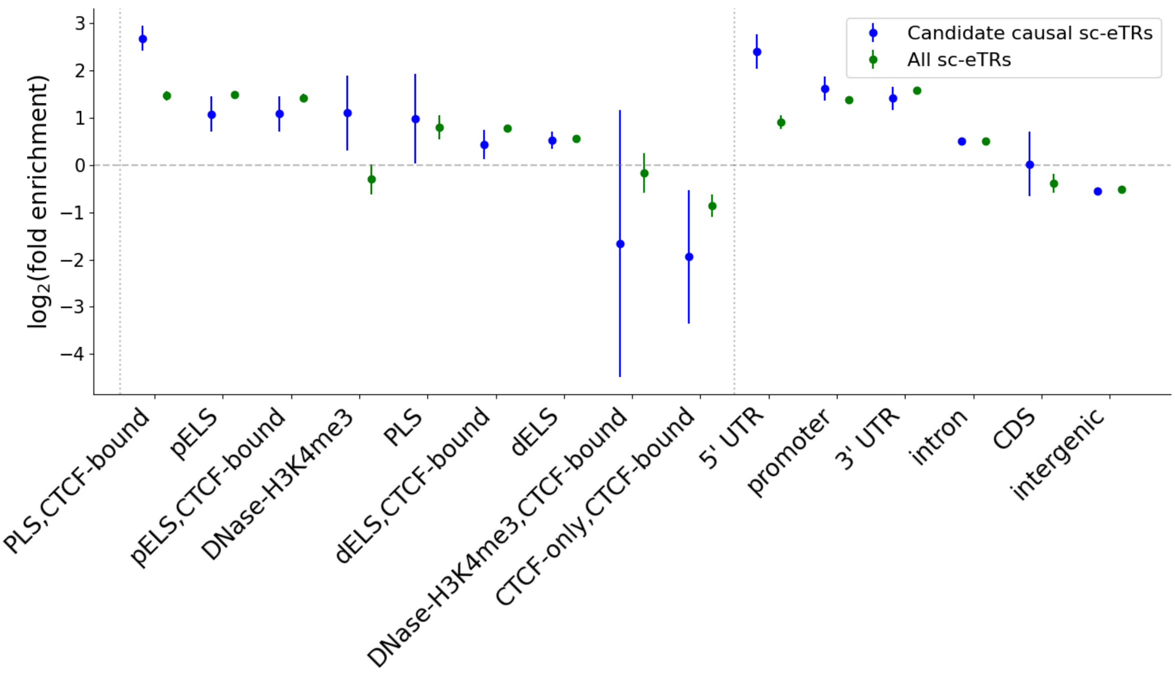

Supplementary Figure 11: Enrichment of sc-eTRs and candidate causal sc-eTRs relative to all TRs genotyped. The y axis denotes $\log_2$fold enrichment, comparing sc-eTRs (FDR <5%) and candidate causal sc-eTRs to all TRs genotyped. Error bars correspond to 95% confidence intervals. ENCODE candidate *cis* regulatory element (cCRE) annotations are abbreviated as follows: promoter-like signature [PLS], proximal enhancer-like signature [pELS], distal enhancer-like signature [dELS], CTCF binding site failing to intersecting another cCRE annotation [CTCF-only].

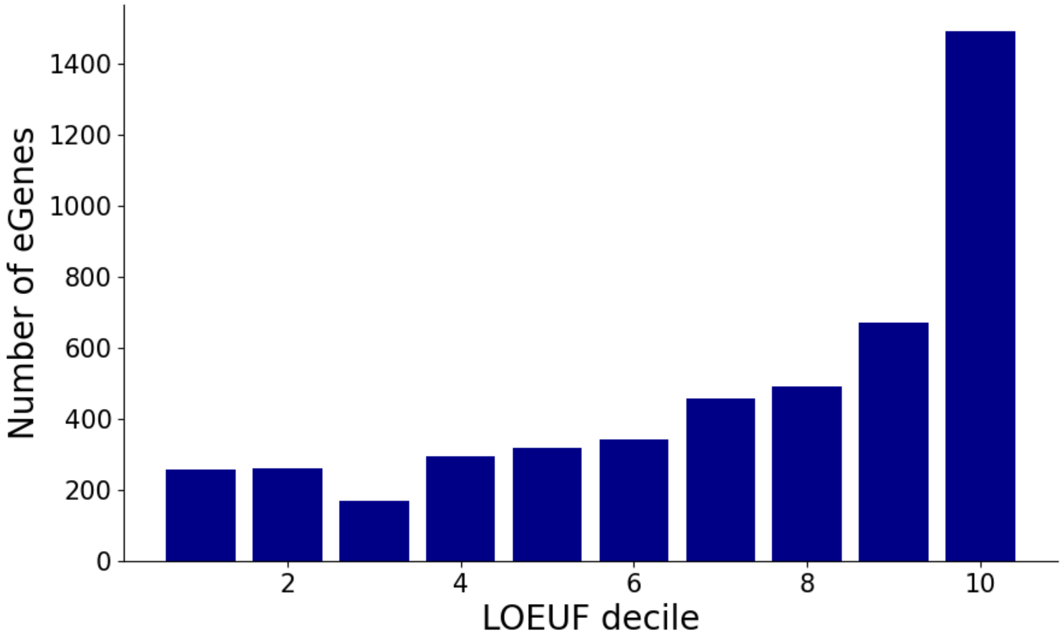

Supplementary Figure 12: **Number of eGenes (associated with fine-mapped sc-eTRs [PIP ≥ 0.7]) by loss-of-function observed/expected upper bound fraction (LOEUF) score decile.** Lower LOEUF deciles correspond to genes with greater constraint.

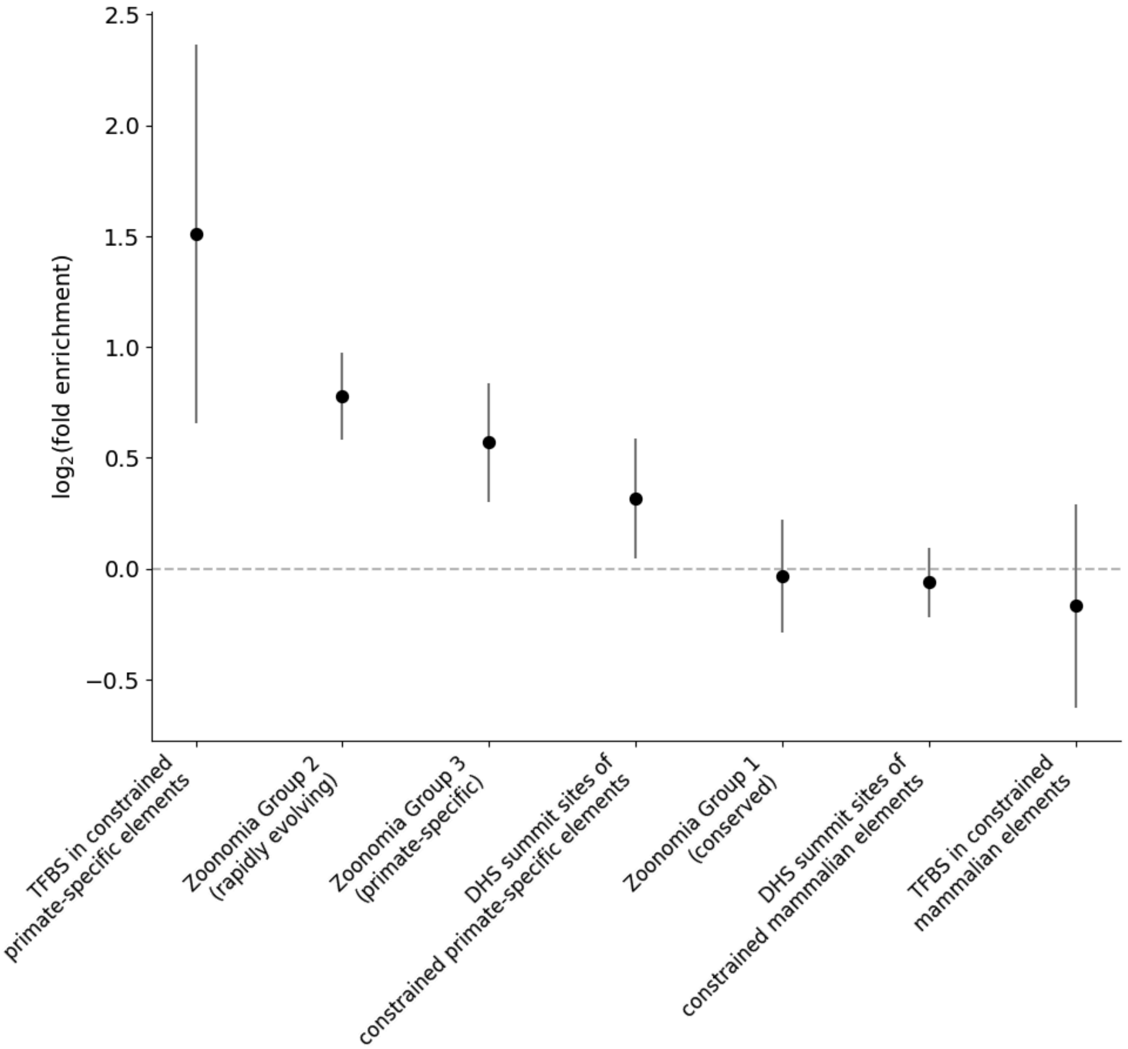

Supplementary Figure 13: Enrichment of candidate causal sc-eTRs relative to all TRs genotyped in primate-specific[17,18] and Zoonomia[19] annotations. The y axis denotes $\log_2$fold enrichment. Error bars correspond to 95% confidence intervals. Abbreviations are as follows: transcription factor binding site [TFBS]; DNase hypersensitivity site +/- 250 bp [DHS].

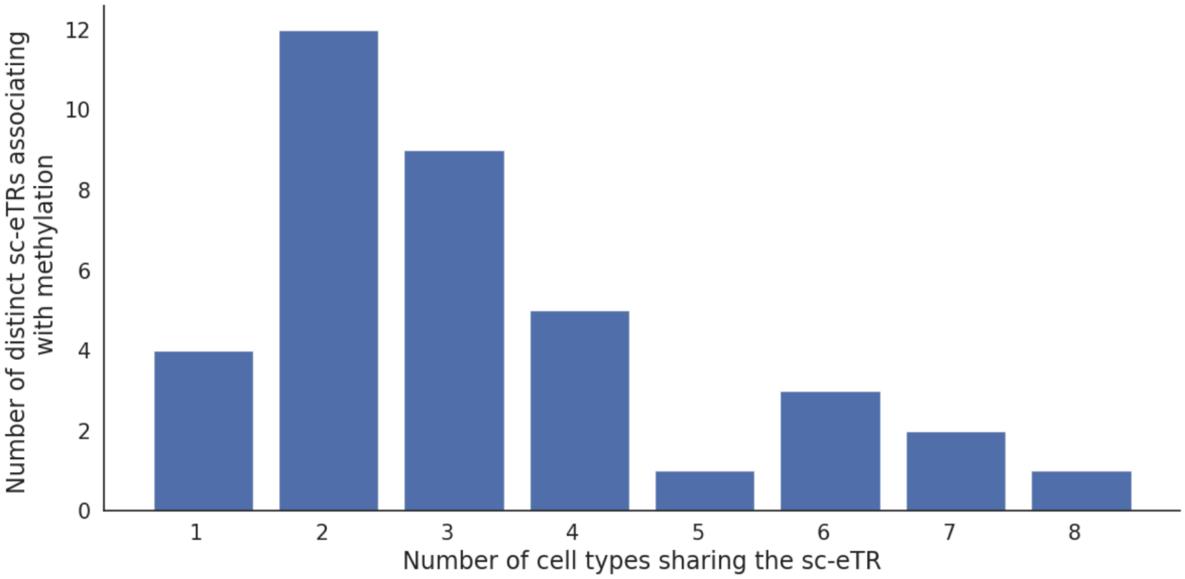

Supplementary Figure 14: Cell type-specificity of sc-eTRs significantly associating with methylation, as measured with PacBio long-read sequencing (Bonferroni-adjusted *P* < 0.05) and significant colocalization (PP H4 ≥ 0.8) with methylation signals derived from a published dataset of SNVs and STRs.

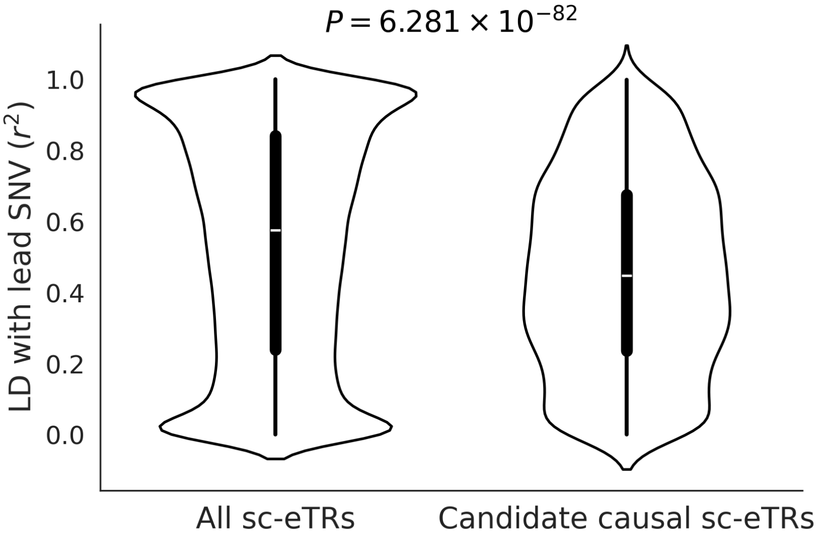

Supplementary Figure 15: Distribution of linkage disequilibrium (LD) with the lead SNV of the respective *cis* window for all sc-eTRs and for candidate causal sc-eTRs. Violin plots summarize the distribution of effect sizes with horizontal lines showing median values and boxes spanning from the 25th (Q1) to the 75th percentile (Q3). Whiskers extend to Q1 – 1.5 x IQR and Q3 + 1.5 x IQR, where IQR is the interquartile range. *Cis* window is the area within +/- 100 kb of the gene body.

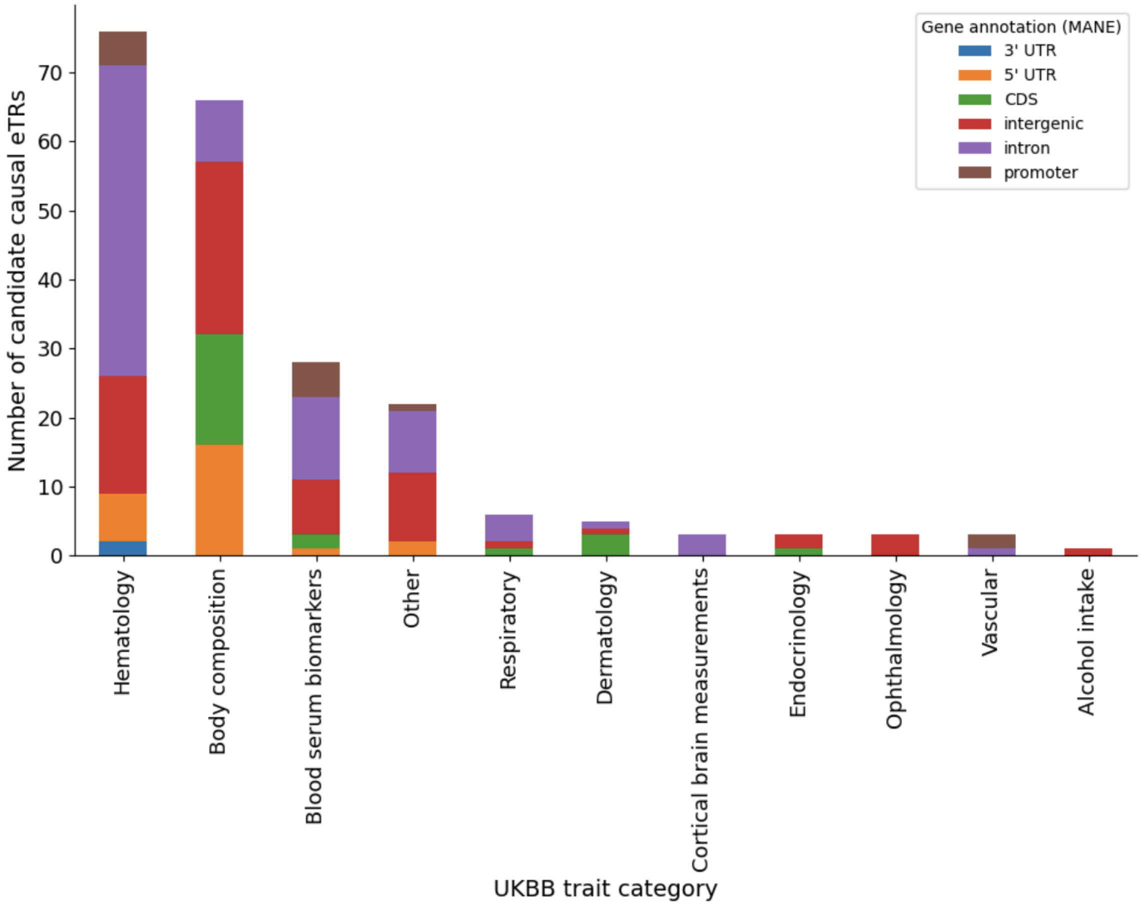

Supplementary Figure 16: Barplot of the number of candidate causal sc-eTRs intersecting significant TR UKBB PheWAS hits, colored by gene annotation.

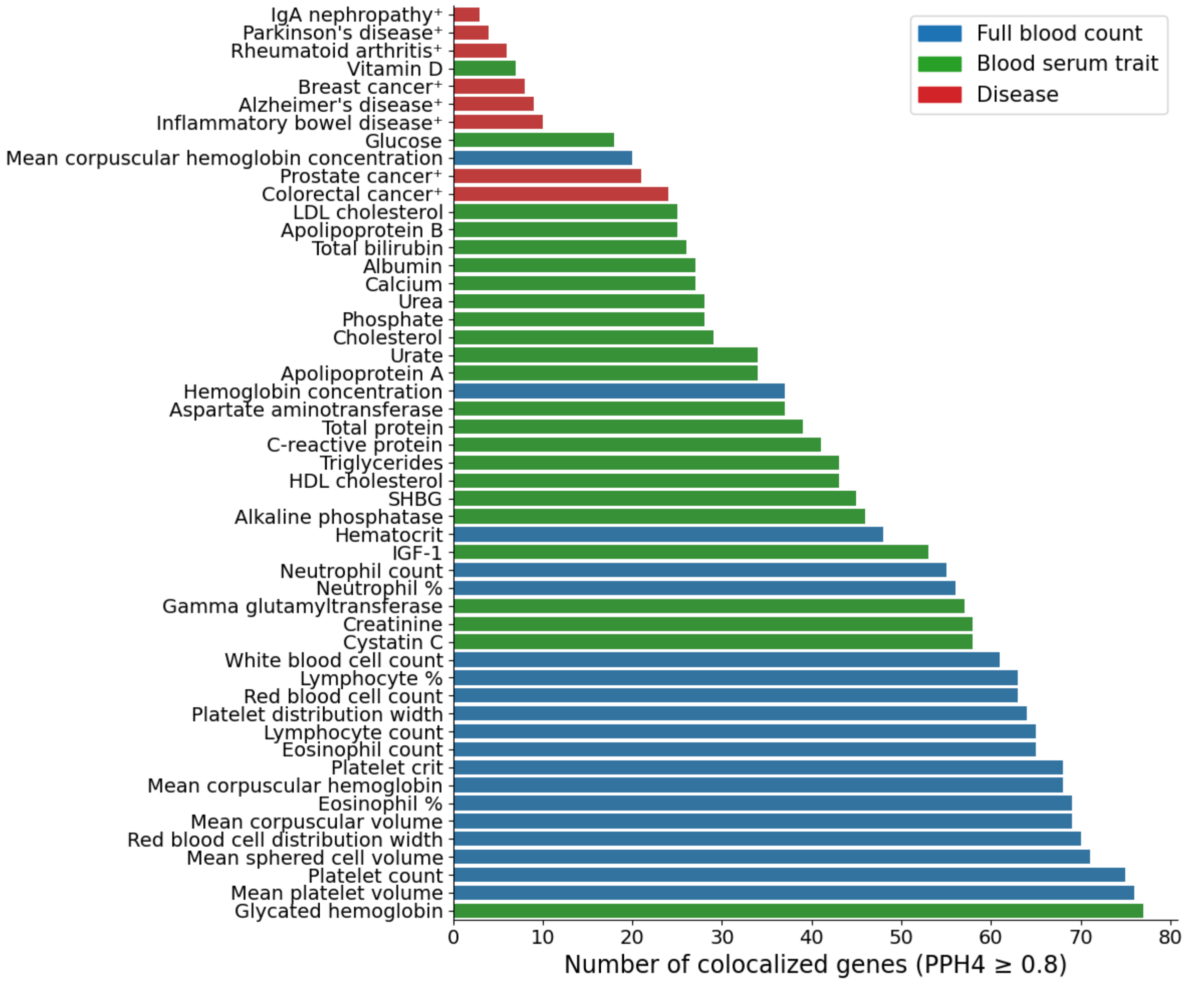

Supplementary Figure 17: Barplot of the number of colocalized genes (PPH4 ≥0.8) containing a candidate causal sc-eTR, per GWAS catalog. Phenotypes marked with † were colocalized using only SNVs while unmarked phenotypes were colocalized using SNVs and imputed TRs.

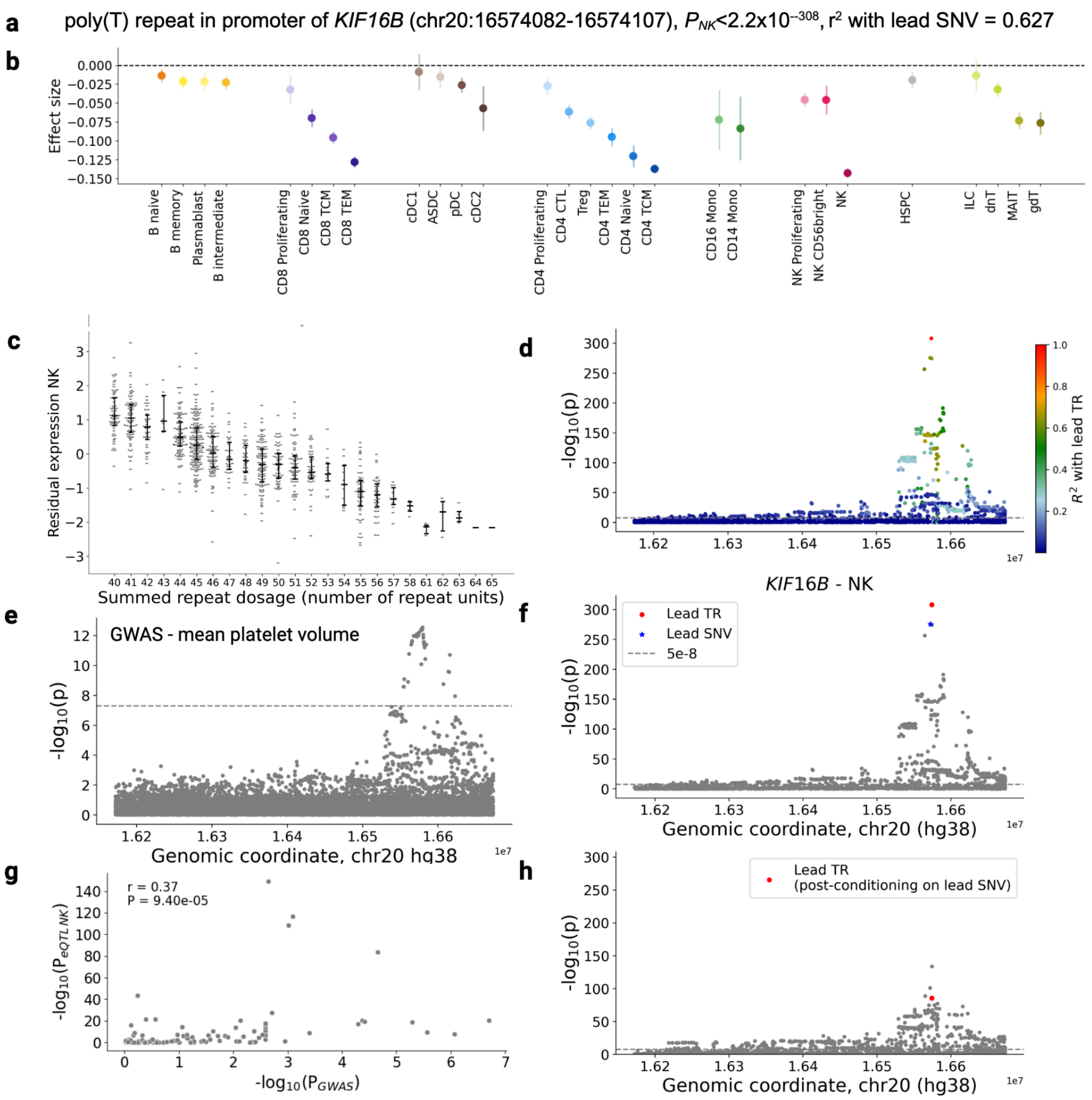

Supplementary Figure 18: poly(T) repeat in the promoter of *KIF16B* colocalizes with GWAS for mean platelet volume. a, Locus details**. b**, Distribution of effect size of the sc-eTR in immune cell types which had sufficient gene expression for association testing (Methods). Error bars correspond to 95% confidence intervals. **c**, Association between repeat length and residual gene expression in NK cells. **d**, sc-eQTL association signals for the candidate causal sc-eTR and other SNVs and TRs within +/- 100kb of the gene body, colored by strength of linkage disequilibrium with the candidate causal TR (R^2). **e,** Mean platelet volume GWAS association signals for the equivalent window (+/- 100kB of the gene body of *KIF16B*). **f**, Association signals for the candidate causal sc-eTR (red circle), the lead eSNV (blue star), and other SNVs and TRs within +/- 100kb of the gene body (gray points). **g**, Association between p-values of overlapping variants in the NK cell sc-eQTL dataset and the mean platelet volume GWAS catalog. **h,** Association signals for SNVs and TRs within +/- 100 kb of the gene body, conditioning each on the genotype of the lead eSNV.

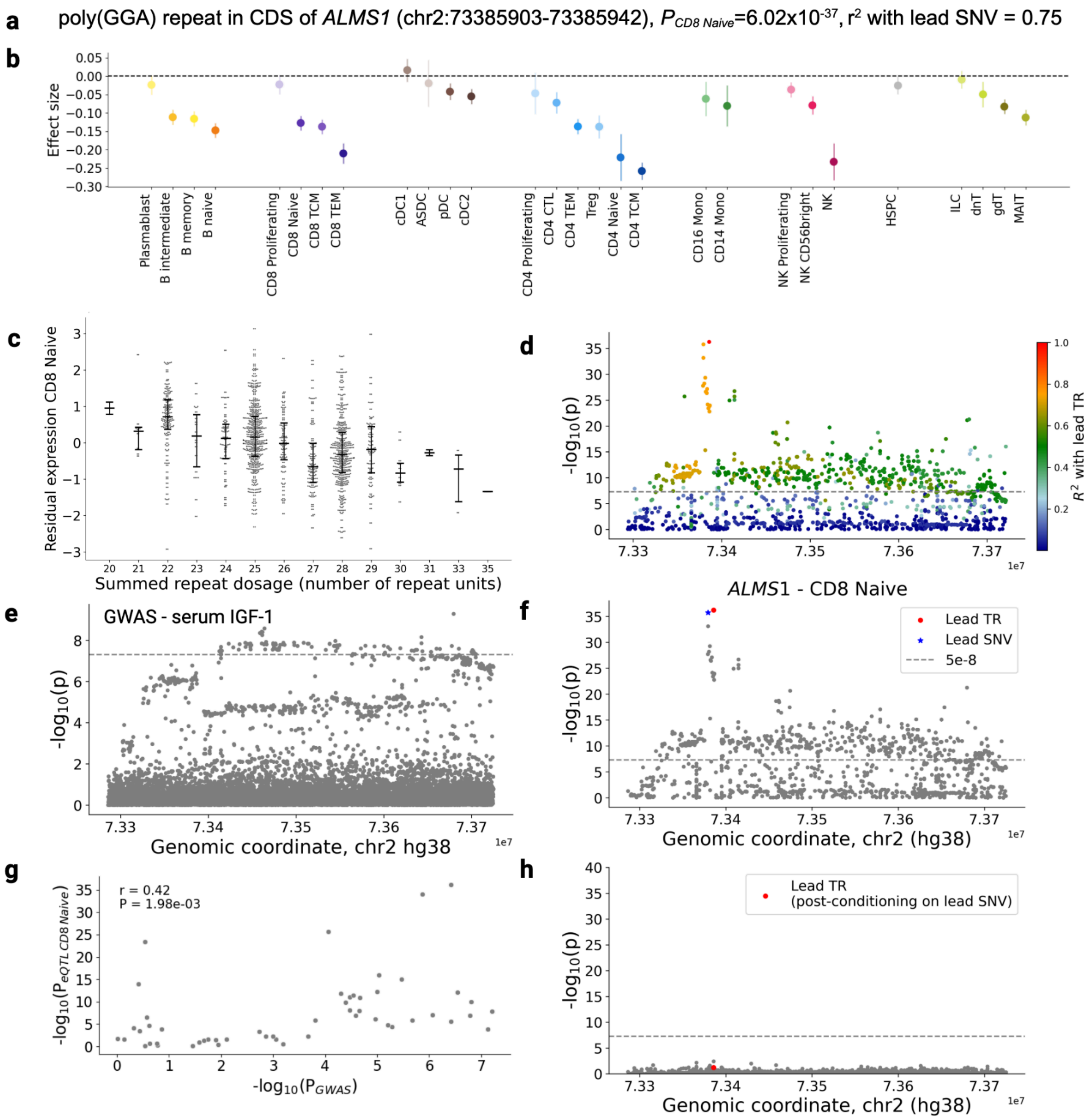

Supplementary Figure 19: poly(GGA) repeat in the CDS of *ALMS1* colocalizes with GWAS for serum IGF-1. a, Locus details. **b**, Distribution of effect size of the sc-eTR in immune cell types which had sufficient gene expression for association testing (Methods). Error bars correspond to 95% confidence intervals. **c**, Association between repeat length and residual gene expression in CD8 naive cells. **d**, sc-eQTL association signals for the candidate causal sc-eTR and other SNVs and TRs within +/- 100kb of the gene body, colored by strength of linkage disequilibrium with the candidate causal TR (R^2). **e,** Mean platelet volume GWAS association signals for the equivalent window (+/- 100kB of the gene body of *ALMS1*). **f**, Association signals for the candidate causal sc-eTR (red circle), the lead eSNV (blue star), and other SNVs and TRs within +/- 100kb of the gene body (gray points). **g**, Association between p-values of overlapping variants in the CD8 naive cell sc-eQTL dataset and the serum IGF-1 GWAS catalog. **h,** Association signals for SNVs and TRs within +/- 100 kb of the gene body, conditioning each on the genotype of the lead eSNV.

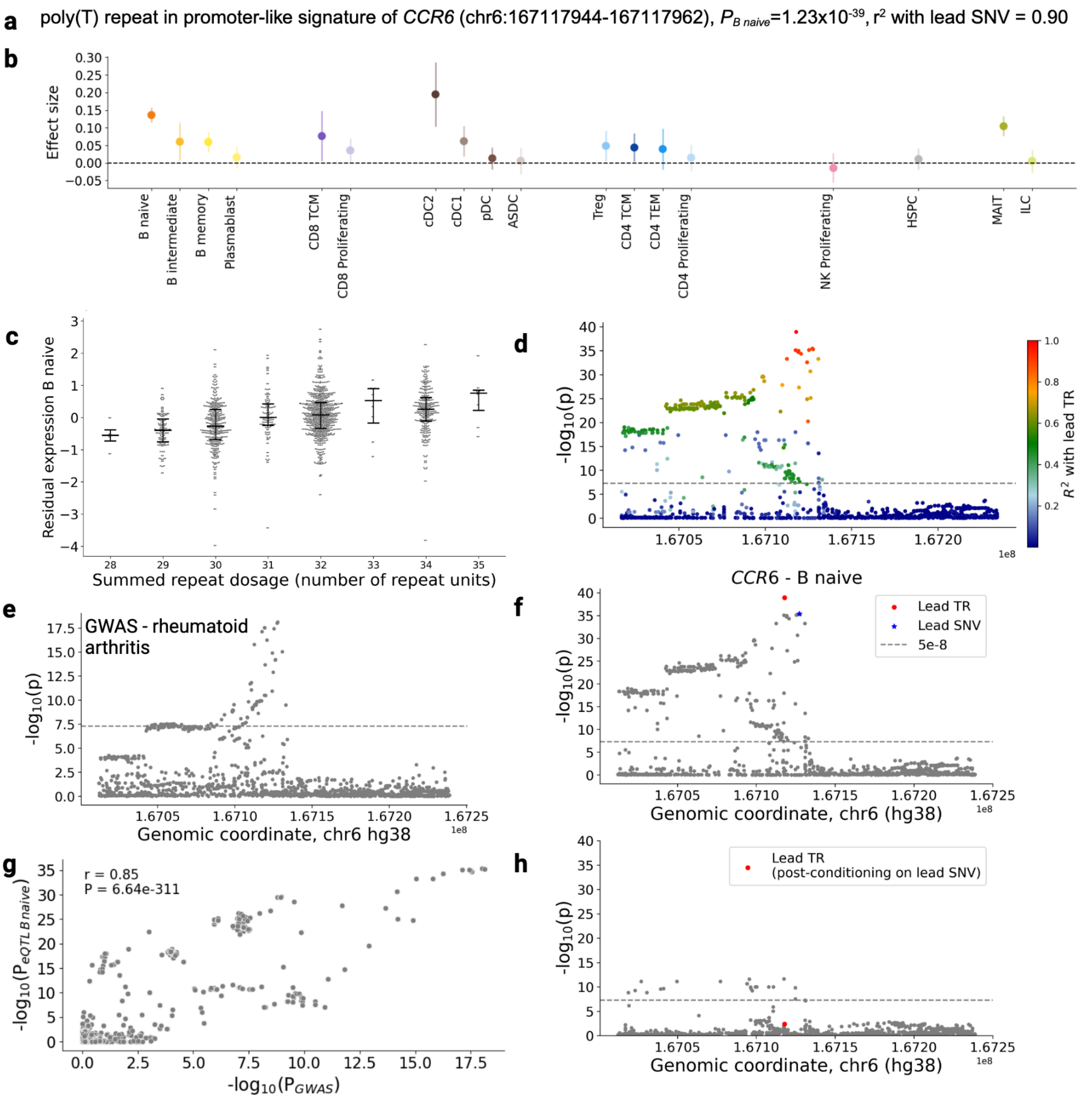

Supplementary Figure 20: poly(T) repeat in promoter-like signature of *CCR6* colocalizes with GWAS for rheumatoid arthritis. a, Locus details. **b**, Distribution of effect size of the sc-eTR in immune cell types which had sufficient gene expression for association testing (Methods). Error bars correspond to 95% confidence intervals. **c**, Association between repeat length and residual gene expression in B naive cells. **d**, sc-eQTL association signals for the candidate causal sc-eTR and other SNVs and TRs within +/- 100kb of the gene body, colored by strength of linkage disequilibrium with the candidate causal TR (R^2). **e,** Rheumatoid arthritis GWAS association signals for the equivalent window (+/- 100kB of the gene body of *CCR6*). **f**, Association signals for the candidate causal sc-eTR (red circle), the lead eSNV (blue star), and other SNVs and TRs within +/- 100kb of the gene body (gray points). **g**, Association between p-values of overlapping variants in the B naive cell sc-eQTL dataset and the rheumatoid arthritis GWAS catalog. **h,** Association signals for SNVs and TRs within +/- 100 kb of the gene body, conditioning each on the genotype of the lead eSNV.

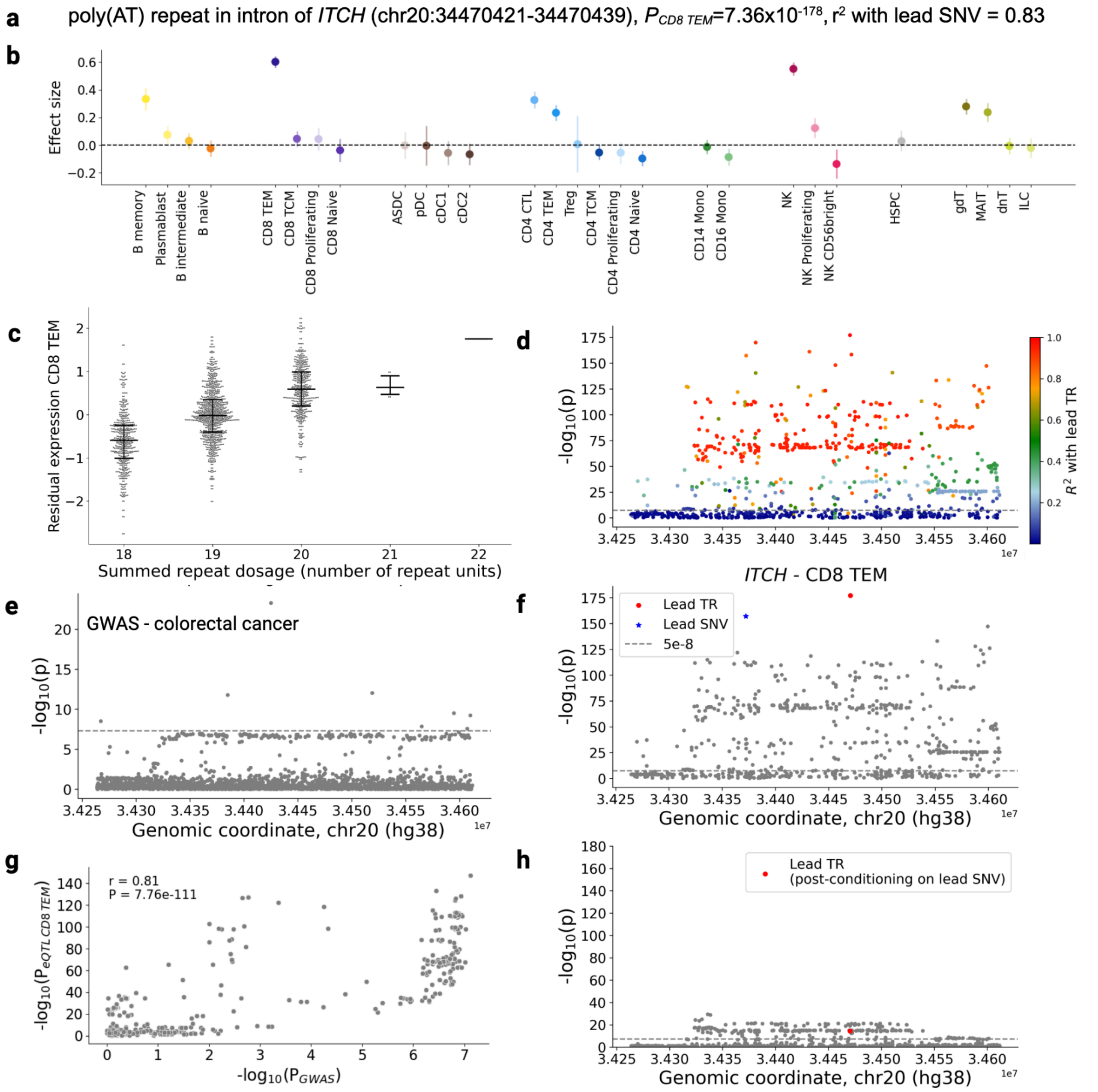

Supplementary Figure 21: poly(AT) repeat in intronic region of *ITCH* colocalizes with GWAS for colorectal cancer. a, Locus details**. b**, Distribution of effect size of the sc-eTR in immune cell types which had sufficient gene expression for association testing (Methods). Error bars correspond to 95% confidence intervals. **c**, Association between repeat length and residual gene expression in $CD8_{TEM}$ cells. **d**, sc-eQTL association signals for the candidate causal sc-eTR and other SNVs and TRs within +/- 100kb of the gene body, colored by strength of linkage disequilibrium with the candidate causal TR (R^2). **e,** Colorectal cancer GWAS association signals for the equivalent window (+/- 100kB of the gene body of *ITCH*). **f**, Association signals for the candidate causal sc-eTR (red circle), the lead eSNV (blue star), and other SNVs and TRs within +/- 100kb of the gene body (gray points). **g**, Association between p-values of overlapping variants in the $CD8_{TEM}$ cell sc-eQTL dataset and the rheumatoid arthritis GWAS catalog. **h,** Association signals for SNVs and TRs within +/- 100 kb of the gene body, conditioning each on the genotype of the lead eSNV.

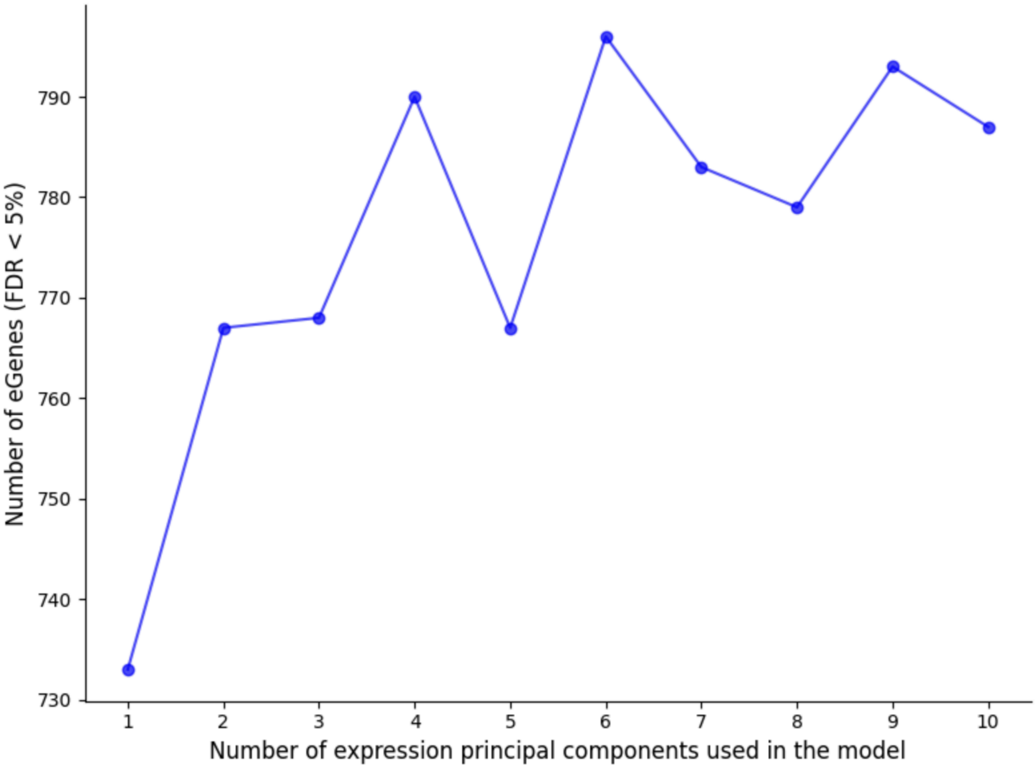

Supplementary Figure 22: Changes in the number of *cis*-eGenes detected with the addition of expression principal components. eGenes (gene-level FDR < 5%) in chromosome 1 were computed for $CD8_{TEM}$ cells after adjusting for a number of expression principal components ranging from 1 to 10. The x-axis shows the number of expression principal components accounted for by the model. The y-axis shows the number of significant eGenes detected at gene-level FDR of 5% using Storey q values[20] on ACAT[21]-corrected p-values. The final model used in the analysis adjusted for 6 expression principal components.

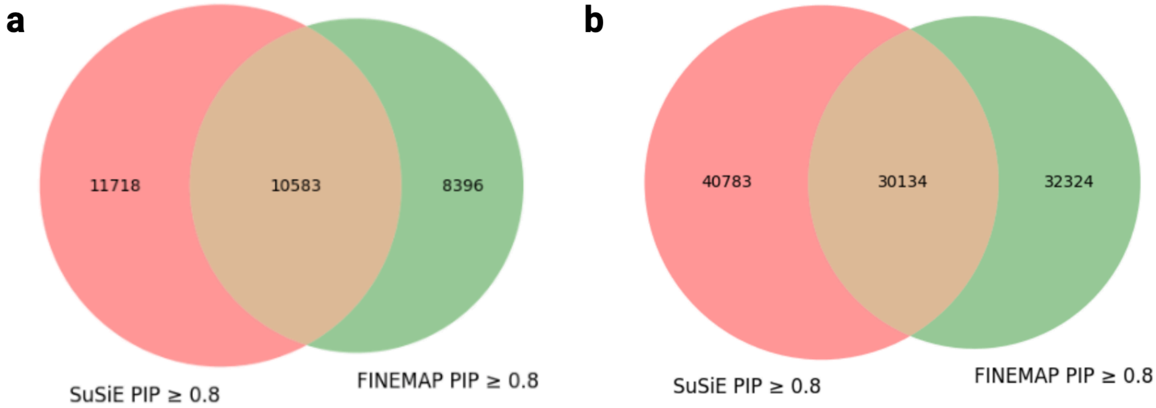

Supplementary Figure 23: **Discordance between SuSIE and FINEMAP fine-mapping tools, with a maximum of 10 credible sets specified.** The number of TRs (a) and SNVs (b) with p-value $<5x10^{-8}$ assigned a posterior inclusion probability ≥ 0.8 by only SuSIE (red), FINEMAP (green), or both (beige).

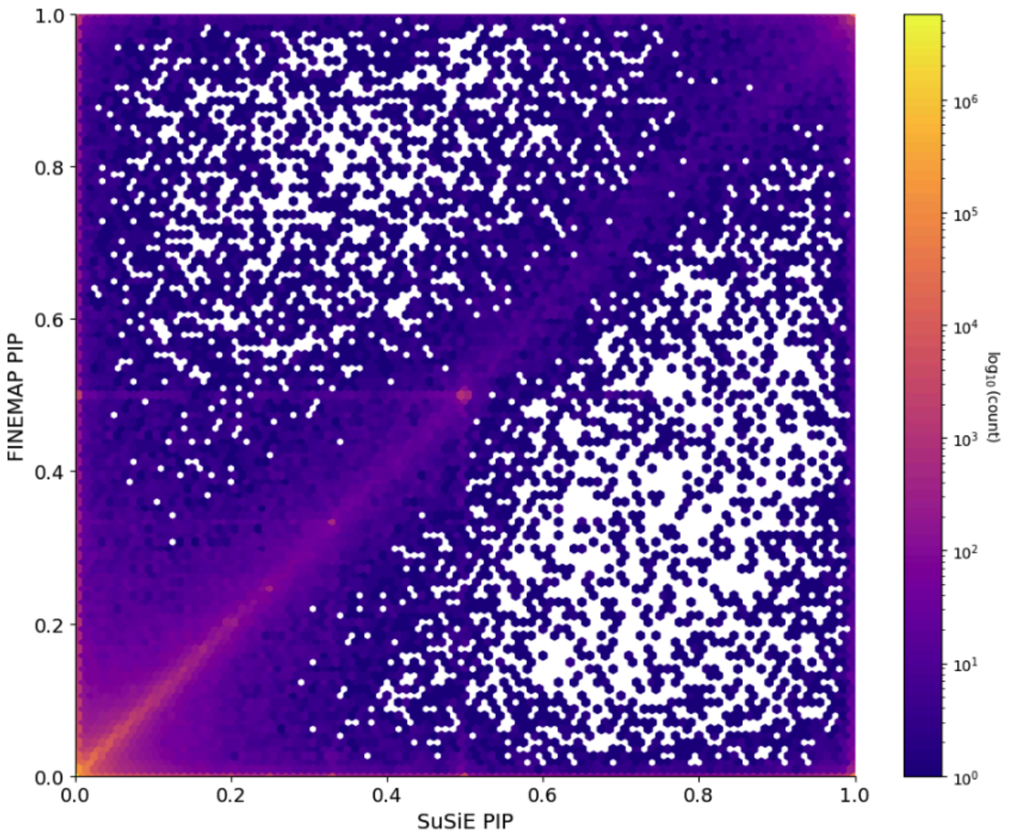

Supplementary Figure 24: Concordance of posterior inclusion probabilities (PIPs) produced by SuSIE and FINEMAP for all TRs and SNVs with p-value < 5x10^{-8}. Density of points was summarized using a hexagonal bin plot with grid size = 100.

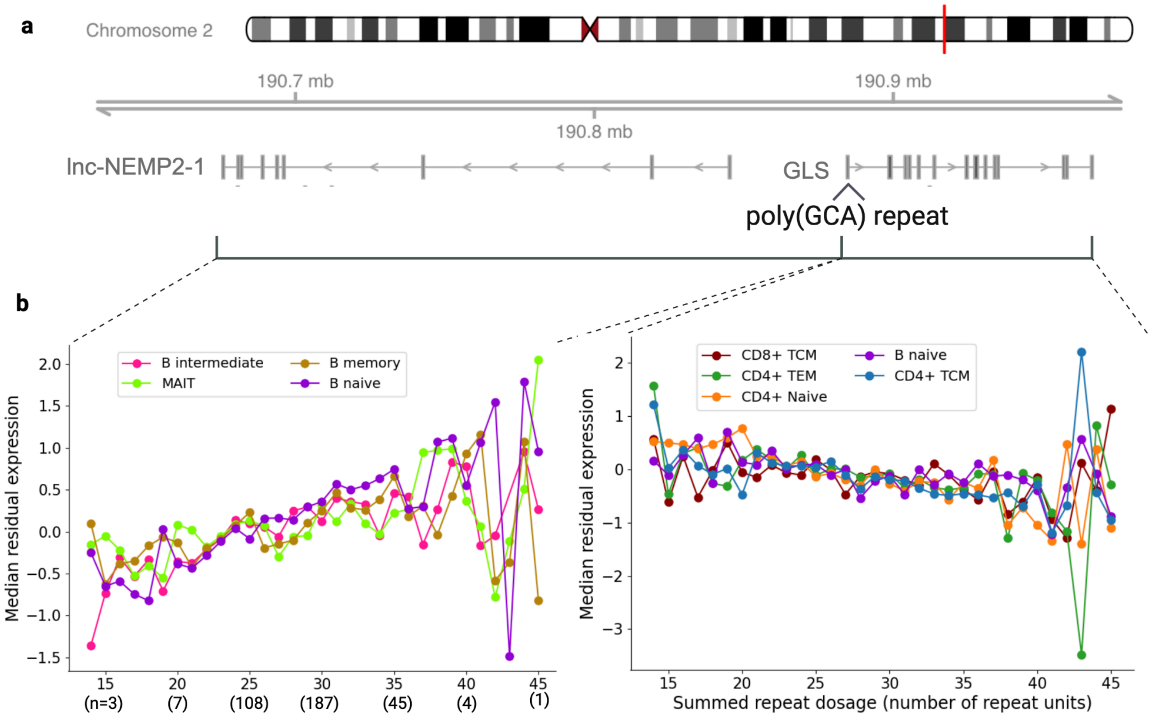

Supplementary Figure 25: Cell type-specific associations of eTRs and expression of *GLS*. **a**, Locus zoom plot of *GLS* and lnc-NEMP2-1 using Gviz[22], loaded with GENCODE v44 track. **b**, Association between repeat length and median residual expression of lnc-NEMP2-1 (left) and *GLS* (right) in cell types where the sc-eTR was considered candidate causal for driving expression of the respective gene.

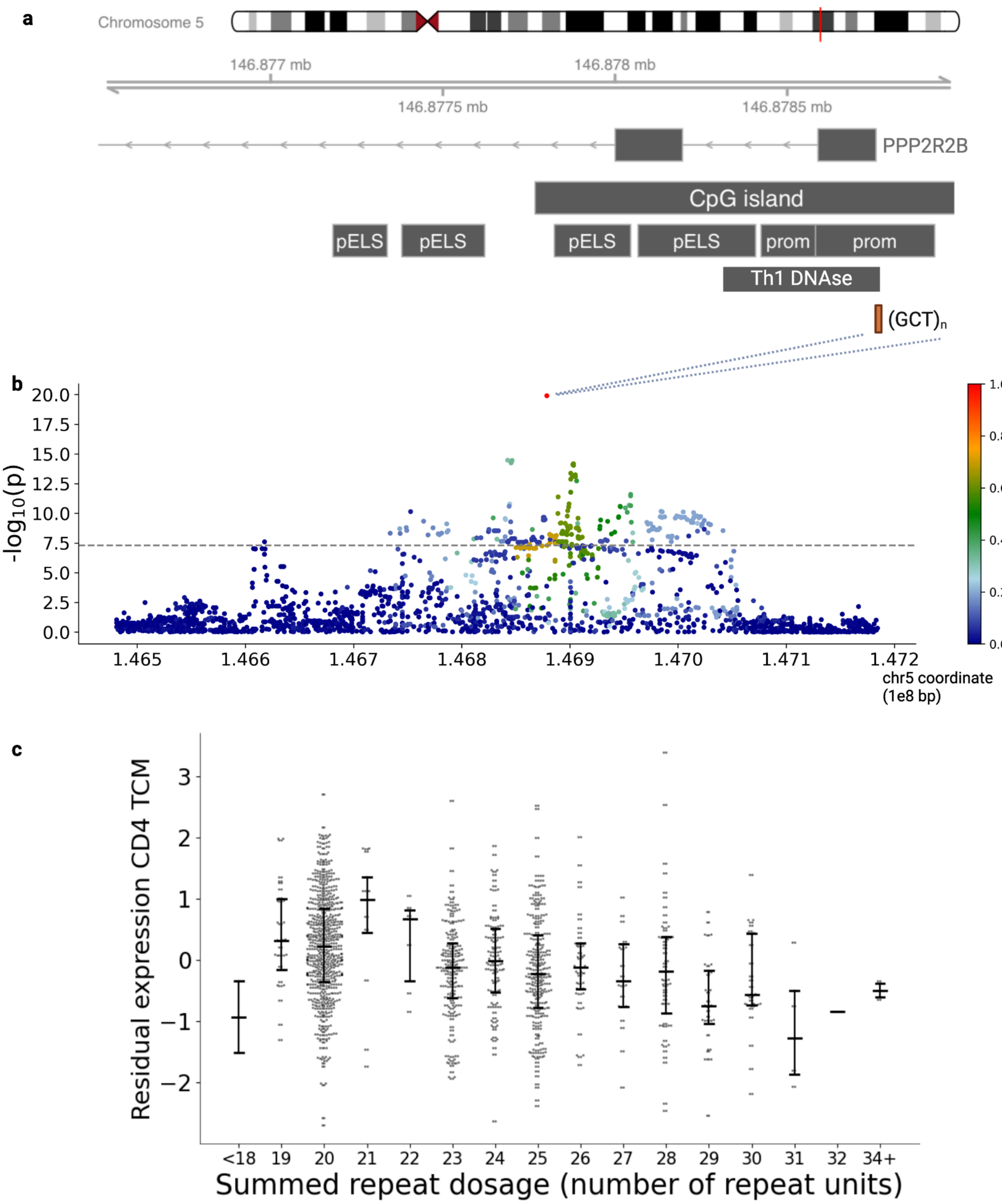

Supplementary Figure 26: poly(GCT) sc-eTR associated with *PPP2R2B* expression. **a**, Locus zoom plot of *PPP2R2B* using Gviz[22], loaded with GENCODE v44 track. **b**, sc-eQTL association signals for the candidate causal sc-eTR (red circle) and other SNVs and TRs within +/- 100kb of the gene body, colored by strength of linkage disequilibrium with the candidate causal TR (R^2). **c**, Association between repeat length and residual gene expression in $CD4_{TCM}$.

Supplementary Tables

All supplementary tables, apart from Supplementary Table 8 below, are provided as supplementary files.

Supplementary Table 8: Candidate causal TRs intersecting known disease-associated loci. Nominal p-value, effect size, and standard error (s.e.) estimates are reported for the cell type with the minimum nominal p-value (in bold). Cell types are listed in order of ascending nominal p-value.

Coordinate (hg38)	Motif	eGene	Cell types	Nominal p-value	Effect size	s.e.
chr2:190880872-190880920	GCA	*GLS*	**$CD4_{TCM}$**, CD4+ naive, $CD4_{TEM}$, B naive, $CD8_{TCM}$	2.6313e-38	-0.040951	0.003164
chr2:190880872-190880920	GCA	lnc-NEMP2-1 (ENSG00000228509)	**B naive**, B memory, B intermediate, MAIT	8.2715e-62	0.058543	0.003529
chr5:146878727-146878757	GCT	*PPP2R2B*	**$CD4_{TCM}$**	1.266796e-20	-0.062647	0.006728
chr10:93702522-93702548	CCG	*FRA10AC1*	**NK**	2.056372e-21	0.049442	0.005203

References

1. Tanudisastro, H. A., Deveson, I. W., Dashnow, H. & MacArthur, D. G. Sequencing and characterizing short tandem repeats in the human genome. *Nat. Rev. Genet.* **25**, 460–475 (2024).
2. Thomas, P. D. *et al.* PANTHER: Making genome-scale phylogenetics accessible to all. *Protein Sci.* **31**, 8–22 (2022).
3. Ashburner, M. *et al.* Gene ontology: tool for the unification of biology. The Gene Ontology Consortium. *Nat. Genet.* **25**, 25–29 (2000).
4. Gene Ontology Consortium *et al.* The gene ontology knowledgebase in 2023. *Genetics* **224**, (2023).
5. Kanai, M. *et al.* Meta-analysis fine-mapping is often miscalibrated at single-variant resolution. *Cell Genom.* **2**, (2022).
6. van Kuilenburg, A. B. P. *et al.* Glutaminase deficiency caused by short tandem repeat expansion in *GLS*. *N. Engl. J. Med.* **380**, 1433–1441 (2019).
7. Calder, P. C. & Yaqoob, P. Glutamine and the immune system. *Amino Acids* **17**, 227–241 (1999).
8. Kumar, M. *et al.* Molecular clues unveiling spinocerebellar ataxia type-12 pathogenesis. *iScience* **27**, 109768 (2024).
9. Madera-Salcedo, I. K. *et al.* PPP2R2B hypermethylation causes acquired apoptosis deficiency in systemic autoimmune diseases. *JCI Insight* **5**, (2019).
10. Crispín, J. C., Apostolidis, S. A., Finnell, M. I. & Tsokos, G. C. Induction of PP2ABβ, a regulator of IL-2 deprivation-induced T-cell apoptosis, is deficient in systemic lupus erythematosus. *Proc. Natl. Acad. Sci. U. S. A.* **108**, 12443–12448 (2011).
11. Thurman, R. E. *et al.* The accessible chromatin landscape of the human genome. *Nature* **489**, 75–82 (2012).
12. Bakhtiari, M. *et al.* Variable number tandem repeats mediate the expression of proximal genes. *Nat. Commun.* **12**, 2075 (2021).
13. Fotsing, S. F. *et al.* The impact of short tandem repeat variation on gene expression. *Nat. Genet.* **51**, 1652–1659 (2019).
14. Gymrek, M. *et al.* Abundant contribution of short tandem repeats to gene expression variation in humans. *Nat. Genet.* **48**, 22–29 (2016).
15. Urbut, S. M., Wang, G., Carbonetto, P. & Stephens, M. Flexible statistical methods for estimating

and testing effects in genomic studies with multiple conditions. *Nat. Genet.* **51**, 187–195 (2019).

16. Alquicira-Hernandez, J., Sathe, A., Ji, H. P., Nguyen, Q. & Powell, J. E. scPred: accurate supervised method for cell-type classification from single-cell RNA-seq data. *Genome Biol.* **20**, 264 (2019).
17. Kuderna, L. F. K. *et al.* A global catalog of whole-genome diversity from 233 primate species. *Science* **380**, 906–913 (2023).
18. Kuderna, L. F. K. *et al.* Identification of constrained sequence elements across 239 primate genomes. *Nature* **625**, 735–742 (2024).
19. Andrews, G. *et al.* Mammalian evolution of human cis-regulatory elements and transcription factor binding sites. *Science* **380**, eabn7930 (2023).
20. Storey, J. D. The positive false discovery rate: a Bayesian interpretation and the q-value. *Ann. Stat.* **31**, 2013–2035 (2003).
21. Liu, Y. *et al.* ACAT: a fast and powerful p value combination method for rare-variant analysis in sequencing studies. *Am. J. Hum. Genet.* **104**, 410–421 (2019).
22. Hahne, F. & Ivanek, R. Visualizing genomic data using Gviz and Bioconductor. *Methods Mol. Biol.* **1418**, 335–351 (2016).
